# IGF2BP3 promotes progression of gallbladder carcinoma by stabilizing KLK5 mRNA in N^6^-methyladenosine-dependent binding

**DOI:** 10.3389/fonc.2022.1035871

**Published:** 2022-10-13

**Authors:** Junzhe Zhang, Kaini Yang, Junfeng Bu, Jiayan Yan, Xiaoqiang Hu, Ke Liu, Si Gao, Shuibin Tang, Lili Gao, Wei Chen

**Affiliations:** ^1^ Department of Biliary-Pancreatic Surgery, Renji Hospital, School of Medicine, Shanghai Jiaotong University, Shanghai, China; ^2^ Department of Liver Surgery, Liver Cancer Institute, Zhongshan Hospital, Fudan University, Key Laboratory of Carcinogenesis and Cancer Invasion, Ministry of Education, Shanghai, China; ^3^ Department of Pathology, Pudong New Area People’s Hospital, Shanghai, China; ^4^ Shanghai Key Laboratory of Biliary Tract Disease, Renji Hospital Affiliated to Shanghai Jiao Tong University School of Medicine, Shanghai, China; ^5^ Shanghai Research Center of Biliary Tract Disease, Renji Hospital Affiliated to Shanghai Jiao Tong University School of Medicine, Shanghai, China

**Keywords:** gallbladder carcinoma, IGF2BP3, KLK5, m6A reader, let-7g-5p

## Abstract

**Background:**

Recent studies have reported that IGF2BP3 is linked to the pathogenesis of various malignancies. Since IGF2BP3 is associated with poor outcomes of gallbladder carcinoma (GBC), we aimed to explore the association between its N^6^-methyladenosine (m6A) RNA methylation and GBC progression.

**Methods:**

Bioinformatic analysis of GSE136982, GSE104165, and RNA-seq was performed. *In vitro* and *in vivo* gain- and loss-of-function assays were done. qPCR, Western blotting, and IHC were conducted in cells or in collected clinical tissue samples. RNA immunoprecipitation, RNA stability measurement, methylated RNA immunoprecipitation, and dual-luciferase reporter assays were performed in this study.

**Results:**

The expression of IGF2BP3 was higher in GBC tissues than in peritumoral tissues. Functions such as cell proliferation and migration, both *in vitro* and *in vivo*, were inhibited by downregulation of IGF2BP3. The analysis of RNA-seq indicated that KLK5 was a downstream target of IGF2BP3. The expression of KLK5 was measured in GBC cells and tumor samples. It was found to be positively correlated with IGF2BP3 level. Upon IGF2BP3 depletion, ectopic expression of KLK5 could rescue cell function in part. Mechanistically, we found that IGF2BP3 directly binds to KLK5 mRNA and regulates its stability in an m6A-dependent manner. As a result, inhibition of KLK5 decreased the expression of PAR2, and deregulated phospho-Akt. Using bioinformatic prediction combined with miRNA microarray analysis, we identified that let-7g-5p is an inhibitor of IGF2BP3, and let-7g-5p expression was negatively correlated with IGF2BP3. Overexpression of let-7g-5p affected the aggressive phenotype of GBC cells by deregulating IGF2BP3, and inhibiting the KLK5/PAR2/AKT axis.

**Conclusions:**

Our data showed that IGF2BP3 is associated with the aggressive phenotype of GBC. Mechanistically, IGF2BP3 activated the PAR2/AKT axis by stabilizing KLK5 mRNA in an m6A-dependent manner. The loss of let-7g-5p enhanced the expression of IGF2BP3 and improved GBC progression. Thus, IGF2BP3 plays a crucial role in GBC, and the let-7g-5p/IGF2BP3/KLK5/PAR2 axis may be a therapeutic target for GBC.

## Introduction

Gallbladder carcinoma (GBC) is a common biliary tract cancer originating from gallbladder epithelium, with an overall 5-year survival rate < 5% (1). Due to late diagnosis and insensitivity to adjuvant chemotherapy, the outcome of GBC has not improved in the past decade, and radical resection remains the only hope for GBC patients (2). Therefore, deciphering the underlying mechanisms of GBC progression, including genetic and epigenetic alterations, and identifying the potential therapeutic targets are particularly significant in GBC research.

Members of the Insulin-like growth factor II messenger RNA binding protein (IGF2BPs) family, including IGF2BP1, IGF2BP2, IGF2BP3, play a crucial role in mediating the transition of IGF2. Accumulating evidence shows that IGF2BPs are closely associated with various pathogenic processes in different types of cancer (3). Previous studies indicated that the oncogenic effect of IGF2BPs relies on the post-transcriptional regulation of mRNAs of numerous oncogenes. Specifically, as an RNA-binding protein (RBP), the interaction between IGF2BPs and the target mRNA depends on recognizing N6-methyladenine (m6A) modified sites in mRNA. IGF2BPs can recruit other regulators to stabilize or degrade mRNA or to activate its translation (4). m6A is the most common target for methylation modification of RNA, and its dysregulation is involved in the pathogenesis of several cancers (5). Lin et al. and Chen et al. demonstrated the complex function of m6A writer METTL3 in the progression or prevention of GBC carcinogenesis (6, 7). Wang et al. demonstrated that translation activation by YTHDF1 is involved in tumorigenesis of colorectal cancer (8). However, whether m6A regulators promote or prevent carcinogenesis is dependent on the type of tumor (9, 10). Thus, we focused on studying the potential effect of IGF2BP3, an RNA m6A modulator, on GBC progression.

In the present study, we found that compared to peritumoral tissues, IGF2BP3 was overexpressed in GBC tissues. IGF2BP3 inhibition prevented the proliferation and migration of GBC. IGF2BP3 directly bound to KLK5 mRNA and stabilized it in an m6A-dependent manner, thereby inducing the AKT pathway *via* PAR2 activation. Interestingly, loss of let-7g-5p in GBC enhanced the expression of IGF2BP3, leading to GBC progression. Taken together, our findings shed light on the involvement of the let-7g-5p/IGF2BP3/KLK5 axis in GBC.

## Methods and materials

### GEO database analysis

Using the R package limma, the GBC mRNA microarray data GSE 136982 was analyzed in order to detect differentially expressed genes between tumor and peritumoral tissues, according to the criteria: |Log_2_FC| ≥ 3 and P < 0.05. The miRNA microarray data GSE104165 was analyzed using GEO2R online tools. Differentially expressed miRNAs were identified between malignant and benign gallbladder tissues, as |Log_2_FC| ≥ 1 and P < 0.05 were set as the thresholds.

### Clinical samples

GBC tumor specimens and paired benign tissues were collected from patients who received radial resection at Renji Hospital, School of Medicine, Shanghai Jiao Tong University between 2019 and 2022. Patients who preoperatively received adjuvant chemoradiotherapy were excluded. Approval of this study was achieved from the ethics committee of Renji Hospital and written informed consent was obtained from all patients. For immunohistochemical staining (IHC), fresh GBC tissue samples were paraffin-embedded after 4% formalin fixation. Each sample was flash-frozen and stored in liquid nitrogen at -80°C for subsequent experiments.

### Cell culture

NOZ and GBC-SD were obtained from the Shanghai Institute for Biological Science, Chinese Academy of Science (Shanghai, China). EHGB-1, OCUG-1 and SGC-996 cell lines were kindly provided by Prof. Yingbin Liu. The culture media used was DMEM/High Glucose (Cytiva, USA) supplemented with fetal bovine serum (Gibco, South American) and 1% Penicillin-Streptomycin. All cell lines were cultivated in a humidified incubator containing 5% CO_2_ at 37°C.

### Quantitative real-time polymerase chain reaction

Total RNA was extracted using TRIzol (Invitrogen) following the manufacturer’s instructions. The mRNA was reverse-transcribed to cDNA by using the Hifair^®^ III 1^st^ Strand cDNA Synthesis SuperMix for qPCR (gDNA digester plus) kit (Yeason, Shanghai). The reverse transcription of miRNA was conducted using miRNA 1^st^ Strand cDNA Synthesis Kit (Vazyme Biotech co., Ltd, Nanjing, China). Quantitative real-time polymerase chain reaction (qPCR) assay was performed to analyze the level of gene expression. Details on the primers used are presented in **Supplementary Materials**.

### Plasmids and siRNA

The pLKO.1 vector was used to generate shRNA (Hanyin biotechnology, Shanghai). The 21 bp sequence of shIGF2BP3 used was CGGTGAATGAACTTCAGAATT (sh1, human) and GCAGGAATTGACGCTGTATAA (sh2, human), respectively. For shMETTL3 plasmid, the 21bp sequence was GCCAAGGAACAATCCATTGTT (human). Scrambled shRNA was used as a negative control. The PLVX-CMV-MCS-3xFlag-PGK-Puro vector (Hanyin biotechnology, Shanghai) was used to overexpress human IGF2BP3 and KLK5, and the empty vector (EV) was used as the control. PLVX-IGF2BP3-3xFlag and PLVX-KLK5 were constructed. The PCR primers used in plasmid construction are listed in the **Supplementary Materials**. The has-let-7g-5p mimics (F: UGAGGUAGUAGUUUGUACAGUU, R: CUGUACAAACUACUACCUCAUU) and mimics NC (F: UCACAACCUCCUAGAAAGAGUAGA, R: UCUACUCUUUCUAGGAGGUUGUGA), were purchased from TsingkeBiotechnologyCo., Ltd (Beijing, China).

### Cell transfection and construction of stable cell lines

The miRNA mimics were diluted to a concentration of 100nM. Cells cultured to 30%~50% density in a 6-well plate were transfected with a mix comprising 2.5μl miRNA mimics (or 2 μg plasmids), and 5μl Lipofectamine 2000 (Invitrogen, California, USA) in 100μl Opti-MEM (Gbico, USA). After 8h, the culture medium was refreshed, and subsequent experiments were conducted after a 40-hour interval.

Lentivirus generated from 293T cells was used to construct stable transfected GBC cell lines. In brief, 293T cells with a density of 30%-50% cultured in a 6cm plate were transfected with a mixture including 1 μg pMD2.G, 2μg pSPAX2, 3μg lentivirus plasmids and 10 μl PEI in 200μl Opti-MEM. After 48h, the culture supernatant was filtered with a 0.22 μm filter (Millex-GP, Millipore, USA) before collection. Further, 1ml lentivirus supernatant supplemented with 1ml fresh culture medium was transfected into GBC cell lines in 30%-50% density with Polybrene (10μg/ml). After 48h, fresh culture medium (5ug/ml puromycin) was used to screen for the stably transfected cells.

### Tumor xenograft assay

Our study protocol of animal experiments was approved by the Animal Ethics Committee of Renji Hospital. BALB/c nude mice (5-6 weeks, 18-20g) were housed under specific pathogen-free conditions. The stable shIGF2BP3 or the corresponding control group of NOZ cells (1 ×10^7^/mice/100μl PBS+10% Matrgel (Corning, California)) were subcutaneously transplanted into each mouse. The mice were monitored daily, and the volume of the tumor was calculated using the formula: (length × width^2^)/2. After 15 days, the mice were euthanized and the xenograft specimens were collected by surgical resection.

### Western blot

RIPA buffer (with PMSF and phosphatase inhibitor) was used to extract whole-cell lysates. Briefly, equal amounts of protein samples were separated *via* SDS-PAGE and transferred to polyvinylidene fluoride membranes. The targeted proteins were detected using different antibodies, including GAPDH (1:1000, Proteintech, USA), IGF2BP3 (1:1000, Proteintech, USA), AKT1 (1:1000, Cell Signaling Technology, USA), P-AKT1(ser473) (1:1000, Cell Signaling Technology, USA), KLK5 (1:1000, #abs136657, Abisin, Shanghai), PAR2 (1:1000, Abclonal Technology Co., Ltd.) and METTL3 (1:1000, Abclonal Technology Co., Ltd.).

### Immunohistochemistry

Immunohistochemistry (IHC) staining was conducted to evaluate the level of IGF2BP3 and Ki-67 expression in histological sections. The detailed protocol has been previously published (11).The intensity of staining in the sections was assessed according to the following criteria: 0, negative; 1, weak positive; 2, moderate positive; 3, strong positive. Depending on the extent of staining, different scores were given as follows: 0, 0% stained; 1, 1–25% stained; 2, 26–50% stained; 3, 51–75% stained; 4, 76~100% stained. The final score for each section was determined by multiplying the intensity scores with the score for the extent of staining. Samples were classified into four grades: 0, negative (-); 1–3, low staining (+); 4–6, medium staining (++); 6–12, high staining (+++).

### Cell growth assay

For CCK8 assays, 1000 cells per well were seeded into 96-well plate. Cell numbers were detected for 5 days using Cell Counting Kit-8 (Yeason, China). 10 μl of CCK-8 reagent mixed with 90μl fresh culture medium was added into the well, and after a 2h incubation, the absorbance was recorded at 450nm using a microplate reader.

1000 cells were resuspended and put into 6-well plate for colony-formation. After 2 weeks, cells were fixed with 4% paraformaldehyde for 10 minutes followed by staining with 0.1% Crystal Violet for 10 minutes. Thereafter, the stained colonies were washed twice and photographed.

To assess DNA synthesis, EdU assay was carried out using BeyoClick™ EdU Cell Proliferation Kit with Alexa Fluor 555 (Beyotime, China) following the manufacturer’s instructions. The detailed methods were previously described (12).

Three independent experiments were performed.

### Transwell assay

To assess cell migration, transwell assays were performed using 8.0 µm pore polycarbonate membrane inserts (Corning Life Science, USA) in 24-well plates. 20000 cells in 200µl serum-free medium were added into the upper chamber, which was placed in a 24-well plate containing 600µl complete medium. After 24 hours, the cells in the lower chamber were treated with 4% paraformaldehyde to fix the cells and 0.1% Crystal Violet for staining. Three random fields were counted.

Three independent experiments were performed.

### RNA-seq

RNA from shCtrl and shIGF2BP3 GBC-SD cells (n=3) was extracted using TRIzol Reagent with the removal of genomic DNA. Next, the RNA quality was determined using Agilent 2100 Bioanalyzer and quantified with an ND-2000 (NanoDrop, Thermo). Each sample containing 1 μg RNA was reverse-transcribed into cDNA to establish the sequencing library. The detailed sequencing methods have been reported previously (13).

### Dual-luciferase reporter assay

The pGL6-miR vector (D2106, Beyotime) was used to constructed a reporter plasmid. The putative sites of let-7g-5p binding to the 3’UTR of IGF2BP3 were amplified and subcloned into a pGL6-miR vector plasmid to generate an IGF2BP3-3’UTR- wildtype (WT) plasmid. The point-mutant primers were designed to generate IGF2BP3-3’UTR- mutant plasmid using PCR based on WT plasmid. Similarly, the predicted sites of KLK5 mRNA bound by IGF2BP3 were amplified and the corresponding reporter plasmids including wildtype or point-mutant sequence were constructed following the same methods.

293T cells were cultured in a 12-well plate for 24 hours. Thereafter, pRL plasmids (100ng) and pGL6-control or other constructed pGL6-miR plasmids (1000 ng) complemented with Lipofectamine 2000 were transfected. Similarly, let-7g-5p mimics (1μl) or PLVX-IGF2BP3(1μg) and the corresponding negative controls were simultaneously transfected. 293T cell extracts were collected after 48 h for analysis using a Dual-luciferase reporter assay kit (Vazyme Biotech co., Ltd, Nanjing, China). The experiments were conducted three times in succession.

The sequences used for construction of reporter plasmids are listed in the **Supplementary Materials**.

### RNA immunoprecipitation

RNA immunoprecipitation (RIP) was performed according to a previously published protocol (14). The antibodies used included anti-Flag (20543-1-AP, Proteintech, USA), anti-IgG (AC005, Abclone, China) and anti-IGF2BP3. Briefly, cell samples were harvested from a 15cm culture plate with a confluence up to 80~90% using 250μl cell lysis buffer. Protein A/G magnetic beads were washed twice and incubated with anti-IgG and anti-Flag at room temporary for 1h. 10μl samples from each lysate were collected and stored in -80°C as RNA input. 100μl of lysate was added to 75μl prepared anti-IgG beads and another 100μl of lysate was added to 75μl prepared anti-Flag beads. These were incubated overnight at 4°C with rotation. The magnetic beads were thereafter washed six times with 1ml wash buffer. 100μl of resuspended beads were collected in new tubes for validation with western blotting, and the remaining 900μl of resuspended beads were collected for RNA isolation using phenol: chloroform: isoamylol (125: 24: 1) reagent. Each RNA sample was reverse-transcribed for subsequent qPCR analysis.

### Methylated RNA immunoprecipitation

Methylated RNA immunoprecipitation (MeRIP) was performed in GBC cells stably transfected with shMETTL3 and shCtrl plasmids. The RNA extracts were transcribed into cDNA for subsequent qPCR analysis and EEF1A was used as a positive control for detecting the regulation of m6A modification by METTL3. The primers for the MeRIP-qPCR assay are presented in **Supplementary Materials**. The protocol followed was based on previous studies (15, 16).

### RNA stability assay

Fresh culture medium containing actinomycin D (10μg/ml) was added to GBC cells cultured in 6-well plate to a confluence of 60%~80%. After actinomycin D treatment, cells were harvested at 0h, 2h, 4h and 6h, respectively. The total RNA was extracted by TRIzol, and reverse transcription was conducted for the subsequent qPCR assay.

### Gel electrophoresis

Gel Electrophoresis was performed to validate the products of RIP-qPCR. Each sample of the RIP-qPCR assay was loaded for separation onto a 2% agarose gel and a DNA ladder (#MD109, Tiangen Biotech, Beijing) was used for indicating the product size. The predicted product size of KLK5 mRNA fragment was 162bp.

### Statistical analysis

The Statistical analysis was conducted with GraphPad Prism software (version 8.0.2, USA). Means and standard derivation (SD) were calculated. The group difference was compared using unpaired or paired student’s t-test. The link within different genes was investigated using Pearson correlation analysis. All data were presented as the mean ± (SD). A two-sided P value less than 0.05 was considered statistically significant.

## Results

### Overexpression of IGF2BP3 in GBC tissues

**Figure 1:**
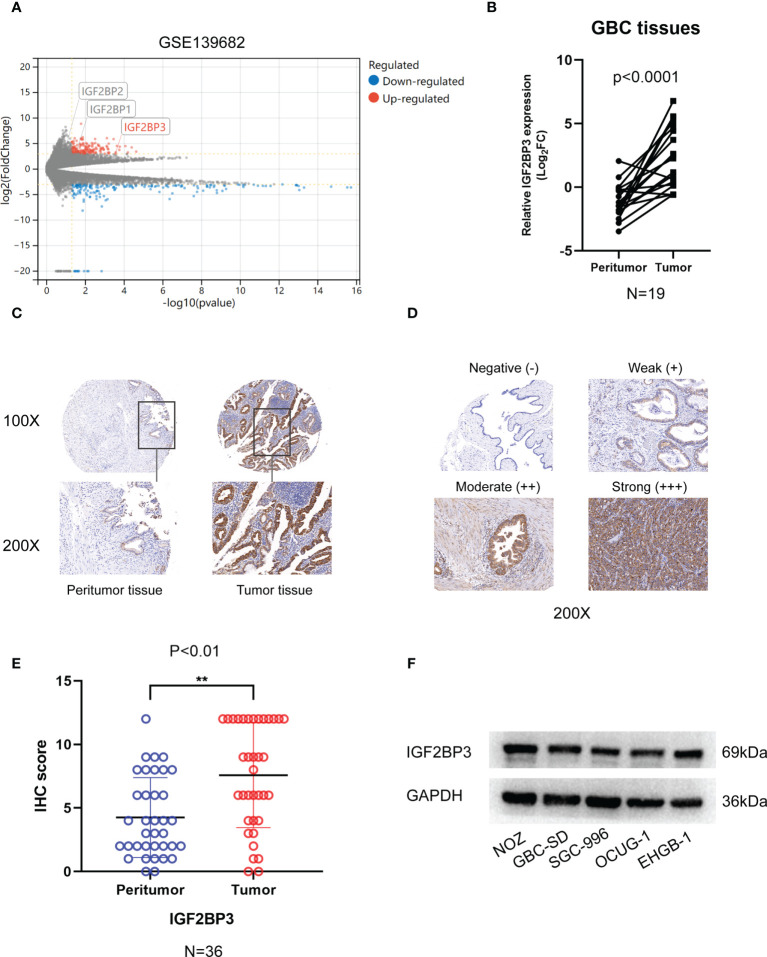
The expression of IGF2BP3 in GBC tissues. **(A)** Volcano plots showed the differentially expressed genes of GBC in GSE139682. **(B)** 19 pairs of GBC tumor tissues and peritumoral tissues were subjected to qPCR to detect the expression levels of IGF2BP3. **(C)** Representative IHC staining images are shown to illustrate differential expression in GBC tissue and paired peritumoral tissue. Bar:100μm. **(D)** Representative image shows negative, weak, moderate, and strong expression of IGF2BP3 in IHC of GBC tissues. The bottom images represent amplified regions shown in square boxes in the top images Scale bar: 100μm. **(E)** IHC scores of peritumoral tissues and GBC tissues. **(F)** The protein expression of IGF2BP3 in GBC cell lines was detected by western blotting. Paired *t* test was used in **(B)** Unpaired *t* test was used in **(E)** Data is shown as mean ± SD, ** P < 0.01.

Analysis of GSE 136982 microarray from the GEO database revealed gene specific expression levels in GBC (|Log_2_FC| ≥ 3, P < 0.05). Only IGF2BP3 of the IGF2BPs family showed a significantly high expression in GBC tumor tissues compared to benign tissues (**Figure 1A**). We examined IGF2BP3 expression in the tissue specimens by qPCR and IHC staining. Using the qPCR assay, we found a significantly higher level of IGF2BP3 mRNA in GBC tumors than in the peritumoral tissues (**Figure 1B**). Further, we performed an IHC staining assay on 36 human GBC tissues and corresponding peritumoral tissues. IGF2BP3 protein was also significantly overexpressed in GBC tumor tissues based on IHC score quantification (**Figures 1C–E**). Western blotting was performed to measure the protein expression in five GBC cell lines. As relatively higher levels were measured in NOZ and GBC-SD and lower levels in OUCG-1, we knocked down IGF2BP3 in NOZ and GBC-SD and enhanced its expression in OCUG-1 for the subsequent experiments (**Figure 1F**).

### Knockdown of IGF2BP3 inhibits GBC proliferation and migration *in vitro*

**Figure 2:**
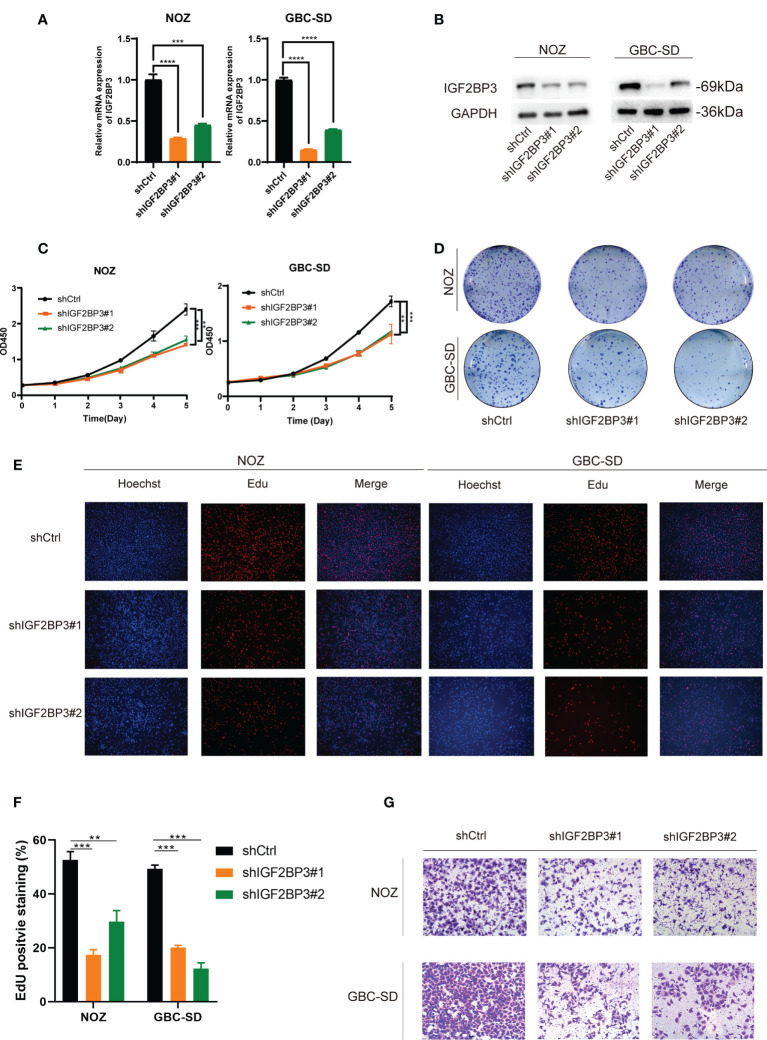
Depletion of IGF2BP3 inhibited GBC cell proliferation and migration. **(A, B)** NOZ and GBC-SD cells were transfected with shRNA to knockdown IGF2BP3. The effect of the transfection was detected by qPCR **(A)** and western blotting **(B)**, respectively. **(C, D)** GBC cell growth was measured by CCK8 **(C)** and colony formation **(D)**. **(E, F)** Cell proliferation was detected by EdU assay using a fluorescence microscope (E, Hoechst (blue), Edu (red), 100X, scale bar: 100μm) and statistical significance was analyzed based on the number of EdU stained cells **(F)**. **(G)** Transwell assays were performed to measure migration ability in treated GBC cells (100X, scale bar: 100μm). Unpaired *t* test was used in **(A, C F)**; data are shown as mean value ± SD; ** P < 0.01, *** P < 0.001 and **** P < 0.0001.

To explore the pathologic role of IGF2BP3 in GBC, we used two shRNAs to silence the IGF2BP3 expression in NOZ and GBC-SD cell lines (**Figures 2A, B**) and overexpressed IGF2BP3 in the OCUG-1 cell line (**Figures S1A, B**). The CCK-8 assay demonstrated that compared with the control groups, IGF2BP3 inhibition significantly decreased cell proliferation in NOZ and GBC-SD (**Figure 2C**). In contrast, IGF2BP3 overexpression enhanced cell growth in OCUG-1 (**Figure S1C**). In parallel, the colony formation assay showed the same patterns as the CCK-8 assay (**Figures 2D** and **S1D**). Furthermore, the EdU assay revealed that depletion of IGF2BP3 attenuated the proliferation of NOZ and GBC-SD, while IGF2BP3 overexpression promoted OCUG-1 growth (**Figures 2E, F** and **S1E**).

Moreover, we performed a transwell assay to explore whether IGF2BP3 influenced cell migration. Compared with the control group, GBC cells transfected with shIGF2BP3 had lower cell migration (**Figures 2G**, **S2A**). OCUG-1 cells transfected with PLVX-IGF2BP3 showed more migration capacity (**Figure S1F**). Taken together, our findings indicated that IGF2BP3 takes part in GBC proliferation and migration *in vitro*.

### IGF2BP3 promoted GBC progression *in vivo*

**Figure 3:**
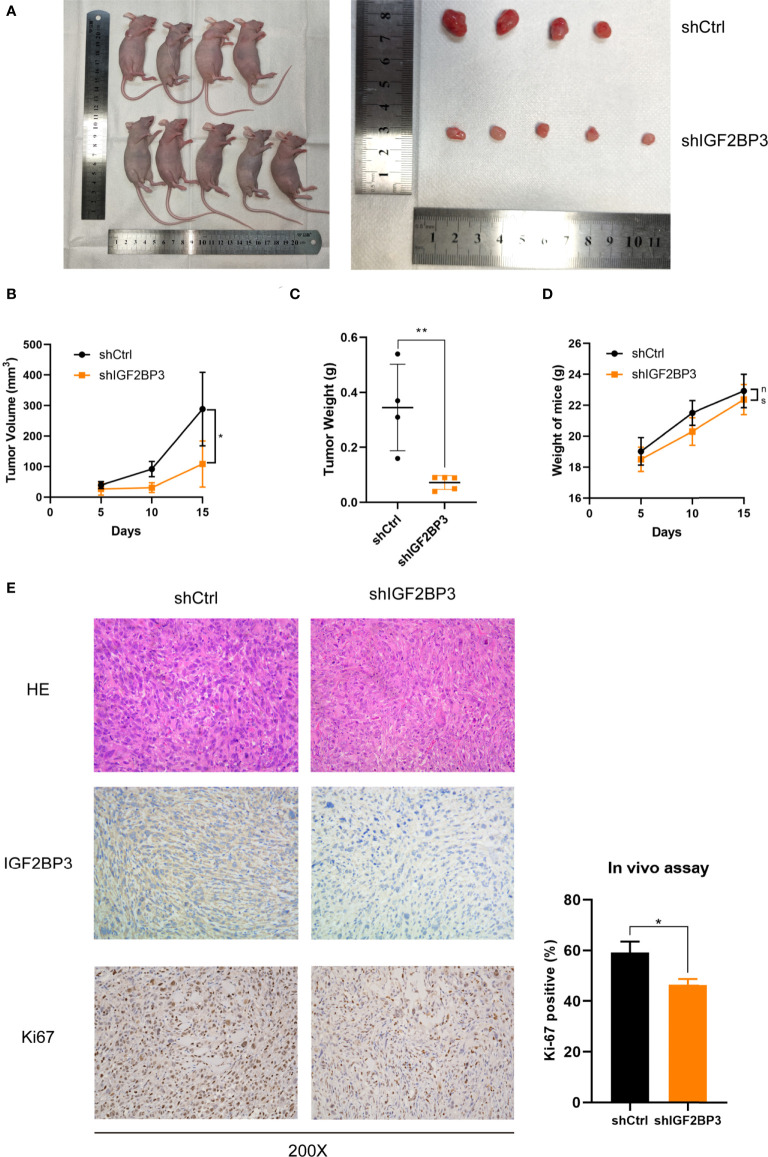
Loss of IGF2BP3 impacted GBC tumor growth *in vivo*. **(A)** NOZ cells from shIGF2BP3 and shCtrl groups were subcutaneously transplanted into nude mice and xenograft tumors were developed. **(B)** Tumor size was measured in order to generate tumor growth curves according to the mean tumor volume. **(C)** Tumor was weighed after surgical resection and shown above. **(D)** The weights of the mice were measured and the weight curves are presented based on the mean weight of each group. **(E)** HE and IHC staining were performed for tumor sections from each group. The Ki-67 positive cells were counted and statistically analyzed using unpaired *t* test. Data is shown as mean ± SD; * P < 0.05 and ** P < 0.01. ns means no significance in statistical analysis.

In order to assess the effect of IGF2BP3 on cell proliferation *in vivo*, we subcutaneously injected NOZ cells with IGF2BP3 depletion into nude mice (**Figure 3A**). Tumor xenograft assays showed that tumorigenesis was suppressed by IGF2BP3 depletion compared with the shCtrl group (**Figures 3A–C**), but no difference in weight change was observed in the two groups (**Figure 3D**). Additionally, results of the IHC showed decreased Ki-67-positive staining in shIGF2BP3 group compared with the control group, confirming the tumorigenicity of IGF2BP3 in GBC (**Figure 3E**). Collectively, IGF2BP3 promoted tumorigenesis *in vivo*.

### KLK5 was regulated by IGF2BP3 in an m6A-dependent manner

**Figure 4:**
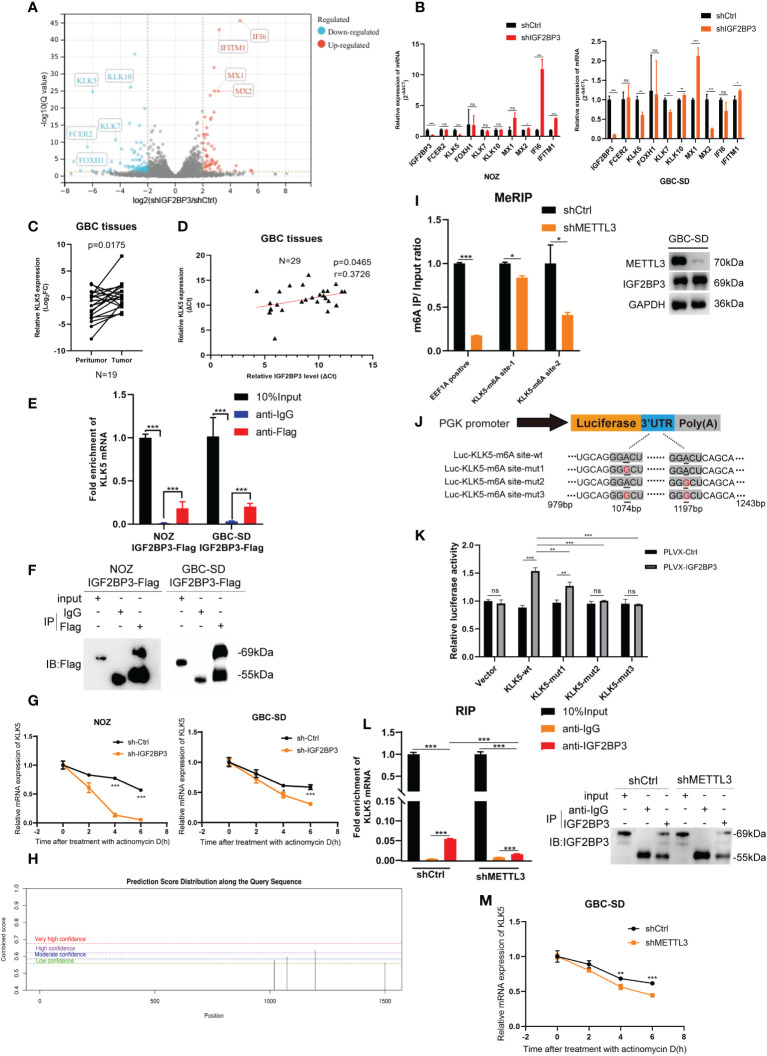
KLK5 was regulated by IGF2BP3 *via* direct binding. **(A)** RNA-seq was performed between shIGF2BP3 and shCtrl cells and the differentially expressed genes are shown in a volcano plot. **(B)** qPCR was performed to verify the downstream target in treated cells of NOZ and GBC based on the results of RNA-seq. **(C)** The KLK5 mRNA levels were measured in 19 pairs of GBC tumor and peritumoral samples. **(D)** The relationship between IGF2BP3 and KLK5 mRNA was analyzed using Pearson correlation tests in 29 GBC tumor tissues. **(E, F)** RIP assay was performed in GBC cells ectopically expressing IGF2BP3-Flag to detect the binding of IGF2BP3 and KLK5 mRNA. Results shown are from qPCR **(E)** and western blotting **(F)**. **(G)** RNA stability tests were performed in GBC cells treated with actinomycin D (10μg/ml), and U6 was used as the endogenous control. **(H)** The putative m6A sites of KLK5 mRNA predicted by SRAMP. **(I)** MeRIP-qPCR was performed in GBC-SD cells with or without METTL3 depletion, and the METTL3 silencing was validated by western blotting (right). The enrichment levels of m6A modified RNA was shown in the left part. **(J)** Luciferase reporter plasmids containing m6A sites of KLK5 mRNA and the point-mutant plasmids were constructed. **(K)** Results of dual-luciferase reporter assay to verify the recognition of IGF2BP3 and m6A sites of KLK5 mRNA. **(L)** RIP assay was performed in GBC-SD cells with or without METTL3 depletion to measure the binding between IGF2BP3 and KLK5 mRNA. Results shown are from qPCR (left) and western blotting (right). **(M)** RNA stability tests were performed in GBC-SD transfected with shMETTL3 or shCtrl after actinomycin D (10μg/ml) treatment, and U6 was used as the endogenous control. Paired t test was used in **(C)** Unpaired t test was used in **(B, E, G, I, K, L, M)**. Data is shown as mean ± SD; * P < 0.05. ** P < 0.01. *** P < 0.001. ns means no significance in statistical analysis.

To explore how IGF2BP3 affects GBC progression, RNA-seq was performed in IGF2BP3 KD and control cells. Differentially expressed genes were screened out when |Log_2_FC|>2 and Q value < 0.05 (adjusted P value) were set as the threshold. Fc epsilon receptor II (FCER2), Kallikrein 5 (KLK5), and Forkhead Box H1 (FOXH1) were down-regulated more than other genes. MX Dynamin Like GTPase 1 (MX1), MX Dynamin Like GTPase 2 (MX2), Interferon Alpha Inducible Protein 6 (IFI6), and Interferon Induced Transmembrane Protein 1 (IFITM1) were up-regulated more than other genes in shIGF2BP3 group. RNA-seq indicated that the gene expression of Kallikrein family including KLK7 and KLK10 also decreased in shIGF2BP3 group (**Figure 4A**). As previously reported, IGF2BP3 can prevent mRNA decay of downstream targets, so we believe that a closer association exists between IGF2BP3 depletion and decreased downstream targets (17). Using a qPCR assay, we found that IGF2BP3 depletion deregulated KLK5 in both NOZ and GBC-SD cells (**Figure 4B**). Next, we measured the mRNA levels of KLK5 in the tumor and peritumoral tissues of human GBC, and investigated the relationship between IGF2BP3 and KLK5 from a clinical perspective. As shown in **Figure 4C**, KLK5 mRNA was upregulated in GBC tumor specimens when compared with paired peritumoral tissues. Additionally, the transcription level of KLK5 positively correlated with IGF2BP3 mRNA levels (**Figure 4D**).

As previously reported, IGF2BP3 is an RNA-binding protein that stabilizes the mRNA of the target gene through a protein-RNA binding mechanism (16). An RIP assay was conducted in both NOZ and GBC cells expressing IGF2BP3-Flag to validate the hypothesis. RIP-qPCR and gel electrophoresis detected the enrichment of KLK5 mRNA in the anti-Flag group, but not in the anti-IgG group (**Figures 4E, F** and **S3A**). In the cells treated with actinomycin D, the stability of KLK5 mRNA significantly decreased when IGF2BP3 was depleted compared with the control group (**Figure 4G**).

Since the IGF2BP family has a higher affinity for the m6A modified regions (RRACH, R=G or A, H= A, C or U) of the target mRNA (16, 17), we hypothesized that the IGF2BP3-KLK5 mRNA binding was regulated by the RNA m6A level of KLK5. The online tool SRAMP (https://wwWCuilab.cn/sramp) was used for predicting m6A modified sites of KLK5 mRNA (NM_012427.5) (18). As shown in **Figure 4H**, the 1072bp~1076bp site (moderate confidence) and 1195bp~1199bp site (high confidence) may be the potential m6A modified regions of KLK5. We performed MeRIP-qPCR in cells with or without METTL3 (an m6A writer) silencing (**Figure 4I**, right). Consequently, the KLK5-m6A site-2 (1195bp~1199bp) showed a greater reduction of m6A level than KLK-m6A-site-1 (**Figure 4I**, left). Next, the dual-luciferase report assay indicated that IGF2BP3 promoted the luciferase activity of KLK5-wt group. This effect could be partly weakened by mut1 and markedly reduced by mut2 and mut3, suggesting that IGF2BP3 protein was most likely to recognize the 1195bp~1199bp site of KLK5 mRNA (**Figures 4J, K**). To further identify whether the changes in m6A level affected the affinity between IGF2BP3 and KLK5 mRNA, we first performed RIP-qPCR in cells with METTL3 depletion and in the control group, and then validated the immunoprecipitation by western blotting (**Figure 4L**, right). As shown in **Figure 4L** (left), the interaction between IGF2BP3 and KLK5 mRNA was significantly abrogated in shMETTL3 group than the shCtrl. The results also showed METTL3-KD accelerated RNA decay of KLK5 mRNA (**Figure 4M**).

Taken together, these findings indicate the positive association between KLK5 and IGF2BP3. Additionally, the recognition of m6A region of KLK5 mRNA by IGF2BP3 impacted the turnover of KLK5 mRNA.

### Inhibition of IGF2BP3 deleted GBC progression by downregulation of the KLK5/PAR2/Akt1 axis

KLK5 is an oncogenic protein involved in inflammation, tumorigenesis, radiotherapy resistance and drug resistance in several cancers (19–25). KLK5 is a serine protease and can activate the transcription of F2R like trypsin receptor 1 (F2RL1), which encodes Protease-Activated Receptor 2 (PAR2) (26–28). As deregulation of the KLK5/PAR2 axis is widely involved in tumor progression, we hypothesized that the AKT pathway that promotes GBC progression, is activated by PAR2 (1, 29–31). As expected, knockdown of IGF2BP3 decreased the expression of KLK5, PAR2, and phospho-AKT1 (Ser473) in NOZ and GBC-SD (**Figure S4A**).

**Figure 5:**
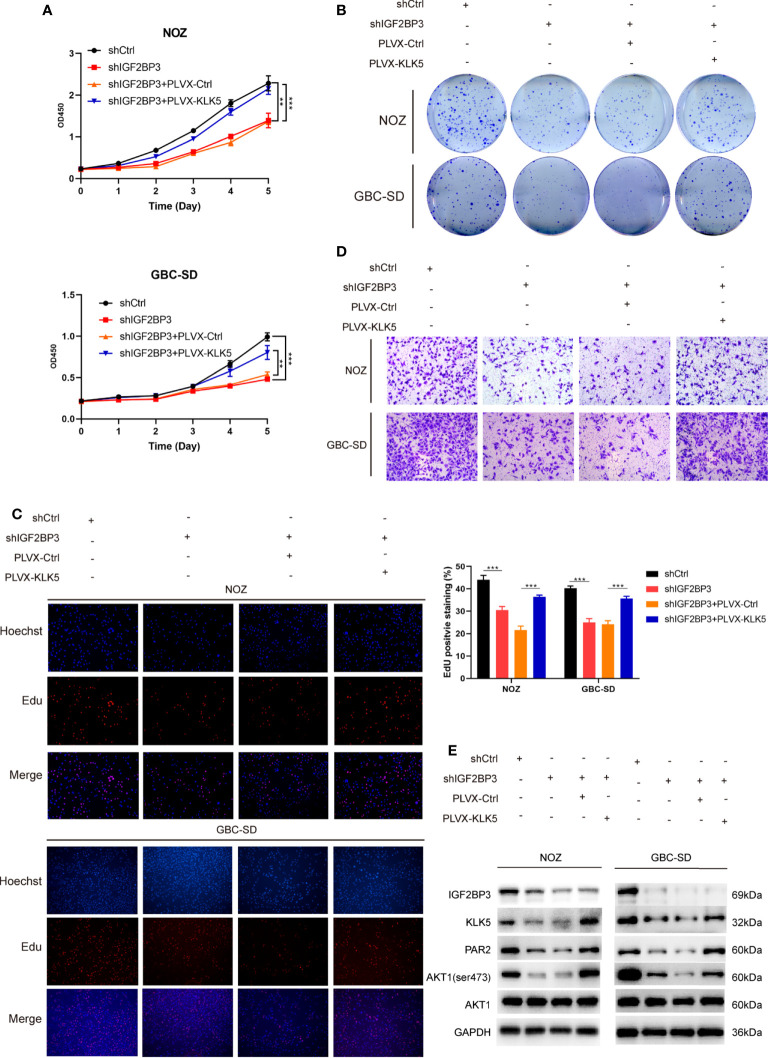
KLK5 rescued the abrogated GBC progression in IGF2BP3-depleted cells. **(A–E)** IGF2BP3-depleted GBC cells were co-transfected with PLVX-KLK5 or control plasmid. Cell growth ability was assessed by CCK8 assay **(A)**, colony formation assay **(B)** and EdU assay (**C**, 100X, scale bar: 100μm). Statistical significance was analyzed based on the number of EdU stained cells (right). **(D)** Transwell assays were conducted to compare the migration ability in each treated group of GBC cells (100X, scale bar: 100μm). **(E)** Western blotting assays showed the expression of IGF2BP3, KLK5, PAR2, p-AKT1 (ser473) and AKT1 in different treatment groups, GAPDH was used as the endogenous control. Unpaired *t* test was used in **(A, C)** Data is shown as mean ± SD; ** P < 0.01 and *** P < 0.001.

Furthermore, in CCK-8, colony formation and EdU assay indicated that KLK5 rescued the cell growth (**Figures 5A–C**) and migration capacity in GBC cells with IGF2BP3 depletion (**Figures 5D** and **S2B**). KLK5 overexpression upregulated PAR2 and phospho-AKT1 (Ser473) in IGF2BP3 deficient cells (**Figure 5E**). These results suggest that IGF2BP3 regulates GBC progression *via* KLK5/PAR2/AKT1 axis.

### let-7g-5p directly targets IGF2BP3 mRNA in GBC

**Figure 6:**
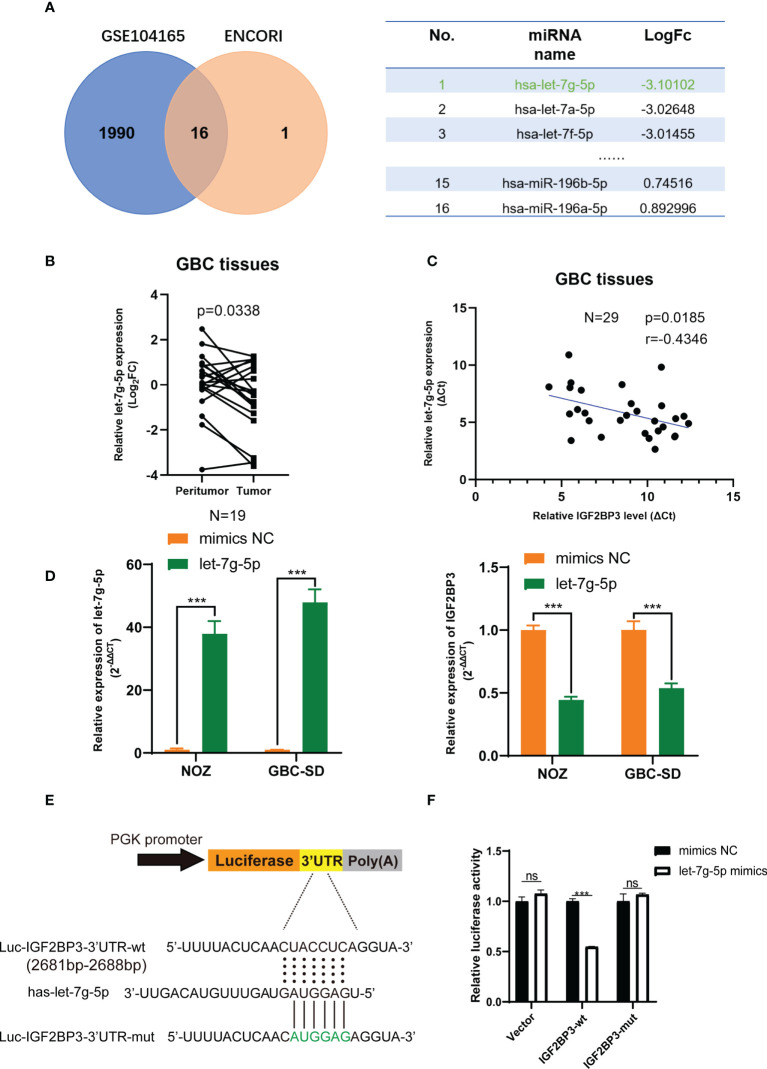
let-7g-5p represses IGF2BP3 by directly targeting its 3’UTR. **(A)** Venn diagram of GSE104165 and the predicted miRNA from ENCORI. The predicted result is listed in the table on the right. **(B)** The expression level of let-7g-5p in 19 pairs of GBC tumor and peritumoral tissues was detected by qPCR. **(C)** Pearson correlation test was conducted to investigate the relationship between let-7g-5p and IGF2BP3. **(D)** The NC and let-7g-5p mimics were respectively transfected into GBC cells to overexpress let-7g-5p. The expression level of let-7g-5p (left) and IGF2BP3 (right) was verified *via* qPCR. **(E)** Luciferase reporter plasmids containing IGF2BP3-3’UTR and the point-mutant plasmids were constructed. **(F)** The result of dual-luciferase reporter assay showed let-7g-5p could silence IGF2BP3 *via* directly interacting with the 3’UTR of IGF2BP3. Paired *t* test was used in **(B)** Unpaired *t* test was used in **(D, F)** Data is shown as mean ± SD; *** P < 0.001. ns means no significance in statistical analysis.

As previously noted, abnormal expression of epigenetic regulators is possibly related to the deregulation of miRNAs (32). The GBC miRNA database GSE104165 (|Log_2_FC| ≥ 1 and P < 0.05) and the online miRNA-mRNA binding prediction tool ENCORI (https://starbase.sysu.edu.cn/) were utilized to identify the potential interaction between miRNAs and IGF2BP3 (Program Number ≥ 5 in ENCORI) (33). Sixteen miRNAs were identified, and let-7g-5p was downregulated in GBC tissues more than other miRNAs (**Figure 6A**). Next, we detected a lower expression level of let-7g-5p in GBC tissues compared with peritumoral tissues (**Figure 6B**). In addition, a negative correlation was found between the transcript level of let-7g-5p and IGF2BP3 in 29 GBC tissues (**Figure 6C**). As expected, IGF2BP3 mRNA expression was decreased in both NOZ and GBC-SD cells with overexpression of let-7g-5p compared with cells transfected with mimics NC (**Figure 6D**).

We performed a luciferase reporter assay to figure out whether IGF2BP3 silencing was post-transcriptionally regulated *via* direct interaction of let-7g-5p. Dual-luciferase reporter plasmids containing IGF2BP3-3’UTR fragment with the let-7g-5p binding region were constructed (**Figure 6E**). Co-transfection of let-7g-5p mimics and the IGF2BP3-3’ UTR-wt vector led to remarkable repression of luciferase activity. This inhibition effect was not observed in the IGF2BP3-3’UTR-mut reporter plasmid transfected group (**Figure 6F**). These findings suggest that let-7g-5p post-transcriptionally inhibits IGF2BP3 through a direct binding mechanism, and loss of let-7g-5p in GBC tissues may be involved in IGF2BP3-mediated tumor progression.

### let-7g-5p inhibited GBC progression by targeting IGF2BP3

**Figure 7:**
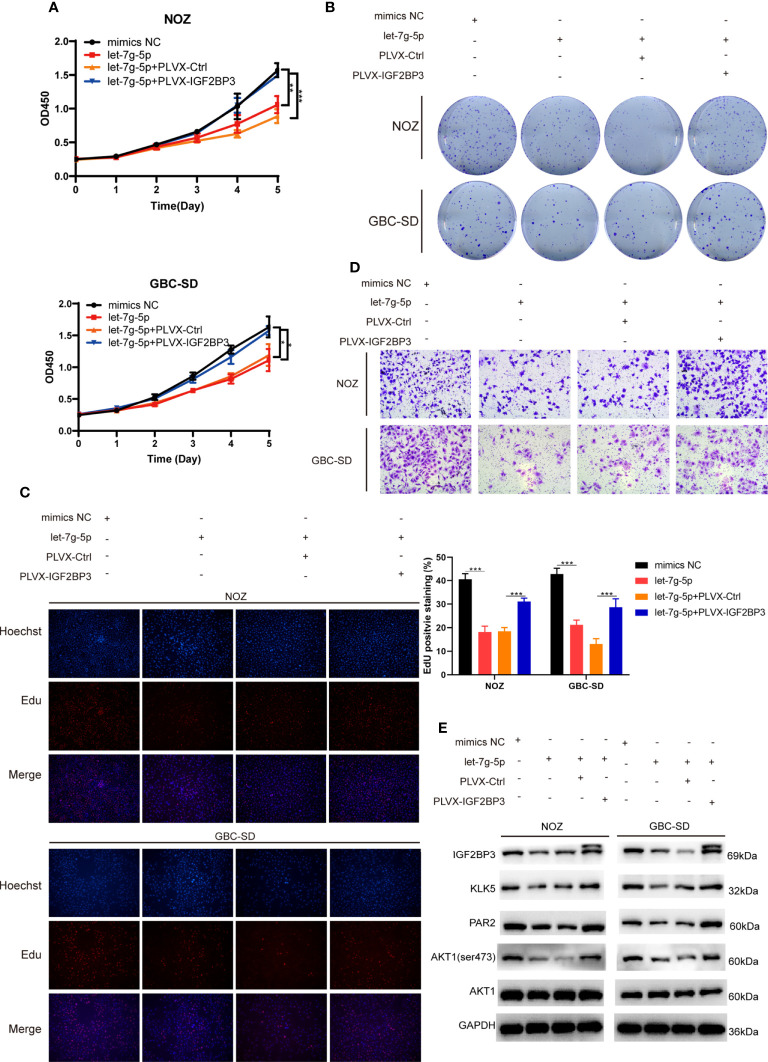
let-7g-5p inhibited GBC progression and was rescued by IGF2BP3 **(A–E)** GBC cells overexpressing let-7g-5p were co-transfected with PLVX-IGF2BP3 or control plasmids. Cell growth capacity was evaluated *via* CCK8 assay **(A)**, colony formation assay **(B)** and EdU assay (**C**,100X, scale bar: 100μm). EdU stained cells was counted and statistical significance was analyzed (right). **(D)** Transwell assays were performed to compare the migration ability in different treatment groups of GBC cells (100X, scale bar: 100μm). **(E)** Western blotting assays showed the expression of targets in the downstream pathway in different treatment groups, GAPDH was used as the endogenous control. Unpaired *t* test was used in **(A, C)** Data is shown as mean ± SD; * P<0.05. ** P < 0.01. *** P < 0.001.

To determine whether let-7g-5p inhibited GBC progression by targeting IGF2BP3, we co-transfected let-5g-5p mimics and PLVX-IGF2BP3 into NOZ and GBC-SD cells. In a cell proliferation assay, we found that let-7g-5p repressed GBC growth, while IGF2BP3 significantly reversed the grow inhibitory effect of let-7g-5p on growth (**Figures 7A–C**). IGF2BP3 also increased the migration capacity of cells overexpressing let-7g-5p (**Figures 7D** and **S2C**). Meanwhile, western blotting indicated that overexpression of let-7g-5p decreased the expression of KLK5, PAR2 and p-AKT (ser473), which were rescued by upregulation of IGF2BP3 (**Figure 7E**).

## Discussion

Recent accumulating evidence has demonstrated that IGF2BP3 is a potential oncogene in many types of cancers. For instance, the overexpression of IGF2BP3 is related to the aggressive phenotype in colon cancer, hepatocellular carcinoma, and nasopharyngeal carcinoma (17, 34, 35). IGF2BP3 also affects tumor immune surveillance and immune infiltration in breast cancer and renal cell carcinoma (36, 37). In addition to solid tumors, ectopic expression of IGF2BP3 modulates hundreds of transcripts through stabilization of mRNA and splicing of pre-mRNA, thus playing an important role in MLL-AF4-mediated leukemogenesis (38). Panebianco et al. (39) demonstrated that the gene fusion of THADA and IGF2BP3 is crucial for IGF2BP3 transcription and IGF1R signaling, promoting tumorigenesis in thyroid cancer. Many studies have shown that dysregulation of non-coding RNAs, including circRNAs, lncRNAs and miRNAs, can influence the oncogenic function of IGF2BP3 (40, 41).

IGF2BPs were primarily recognized as RNA-binding proteins that regulated the fate of RNAs. In 2018, Huang et al. (16) demonstrated that protein-RNA interactions depend on the recognition of m6A modified sites in the target mRNA. Therefore, effects of this interaction can be influenced by m6A modified levels of mRNA. IGF2BPs can recruit other RBPs, such as HuR, to delay RNA decay through direct interaction. IGF2BPs can also protect the target RNA by forming stress granules and activating the translation of mRNA in an m6A-dependent manner (16, 42). N6-methylation modification of RNA is also involved in the development of GBC. METTL3, a crucial m6A writer, is associated with deoxycholic acid-mediated growth inhibition in GBC (6). METTL3 also regulates the aggressiveness of GBC by modulating DUSP5 (7). Kim et al. (43) indicated that high IGF2BP3 expression reflects the aggressive phenotype of GBC and is related to poor prognosis in patients. Thus, it is worth elucidating the epigenetic modifications of IGF2BP3 in GBC progression.

Here, we have reported that IGF2BP3 was found to be significantly overexpressed in GBC tissues *via* qPCR and IHC staining. Using CCK-8, colony formation, EdU assays and xenograft experiments, we found that IGF2BP3 enhanced GBC cell proliferation *in vitro* and *in vivo*. Moreover, transwell assays indicated that the depletion of IGF2BP3 dramatically inhibited cell migration. Through analysis of RNA-seq in shCtrl and shIGF2BP3 cells, we identified KLK5 as a target gene of IGF2BP3. Recently, Zhou et al. (19) reported that the upregulation of KLK5 is associated with aggressive behavior and radioresistance of cervical cancer, leading to local recurrence and metastasis. Tian et al. (20) found a correlation between KLK5 and breast cancer development and COX-2 inhibitor resistance. Previous studies have consistently indicated that KLK5 is a potential serum biomarker for breast cancer (44). Our data has shown that ectopic expression of KLK5 can rescue cell proliferation and migration in GBC cells with suppressed IGF2BP3 levels. Mechanically, IGF2BP3 can directly bind to the m6A modified regions of KLK5 mRNA and increase the half-life of its mRNA. In addition, inhibition of METTL3 depleted the m6A modification level of KLK5 mRNA, which led to its low affinity for IGF2BP3.

KLK5 can promote the expression of F2RL1, the gene coding PAR2 (27). The KLK5/PAR2 axis plays a crucial role in inflammation and carcinogenesis in human skin (21, 24, 26). Recent studies have demonstrated that PAR2 can activate of AKT, probably by inhibiting PTEN (30, 31). Furthermore, the AKT signaling pathway widely participates in several pathogenic processes in developing GBC, such as epithelial-mesenchymal transition, chemoresistance, and immune escape (29, 45–47). We found that loss of IGF2BP3 decreased the expression of KLK5, PAR2, and p-AKT (ser473), which could be reversed by KLK5.

Cancer-related miRNAs are closely associated with tumorigenesis through silencing of the target mRNAs (48). Using the ENCORI tool and miRNA microarray of GBC, we found that let-7g-5p may be the upstream inhibitor of IGF2BP3. The let-7 family was the first known family of miRNAs. Many studies attempted to uncover the mechanisms by which let-7 prevents cancers (49, 50). Lin28 is an oncogenic RBP that abrogates let-7 expression. The Lin28/let-7 pathway is deeply involved in tumorigenesis, cancer immunotherapy, cancer stem cell biology, cell metabolism, metastasis, chemoradiation resistance, etc (51, 52). However, the role of let-7 in GBC needs to be further clarified. Using cell function assays, we uncovered that let-7g-5p inhibits the oncogenic role of IGF2BP3 in tumor progression, and regulates the activity of the KLK5/PAR2/AKT axis in GBC.

In conclusion, through interaction with m6A sites and stabilization of KLK5 mRNA, IGF2BP3 promotes GBC progression *via* the PAR2/AKT axis. In addition, overexpression of let-7g-5p can significantly attenuate the oncogenic function of IGF2BP3 in GBC. Genes in this axis may have great potential to be used as diagnostic biomarkers and therapeutic targets for GBC treatment.

## Data availability statement

The datasets presented in this study can be found in online repositories. The names of the repository/repositories and accession number(s) can be found in the article/**Supplementary Material**.

## Ethics statement

The studies involving human participants were reviewed and approved by ethics committee of Renji Hospital. The patients/participants provided their written informed consent to participate in this study. The animal study was reviewed and approved by Animal Ethics Committee of Renji Hospital.

## Author contributions

JZ, KY, and JB contributed equally to this work and were co-first author of the study. WC and LG contributed to study designation and obtaining funding. JZ, LG, and KY performed the experiments and produced the initial manuscript. JZ, KY, JB, JY, XH, and KL contributed in collection of clinical samples and work discussion. JZ, LG, and WC contributed in data analysis and interpretation. WC, JZ, JB, SG, and ST contributed in statistical analysis and draft revision. All authors contributed to the article and approved the submitted version.

## Funding

This work was supported by grants from Shanghai Shen Kang Development Center (16CR3028A) and the Pudong New Area Science and Technology Development Fund of Shanghai (PKJ2020-Y40).

## Acknowledgments

The authors appreciated the help from Prof. Yingbin Liu from Department of Biliary-pancreatic Surgery, Renji Hospital, School of Medicine, Shanghai Jiao Tong University, Shanghai China, Shanghai Key Laboratory of Biliary Tract Disease, Renji Hospital Affiliated to Shanghai Jiao Tong University School of Medicine, Shanghai 200120, China, Shanghai Research Center of Biliary Tract Disease, Renji Hospital Affiliated to Shanghai Jiao Tong University School of Medicine, Shanghai 200120, China.

## Conflict of interest

The authors declare that the research was conducted in the absence of any commercial or financial relationships that could be construed as a potential conflict of interest.

## Publisher’s note

All claims expressed in this article are solely those of the authors and do not necessarily represent those of their affiliated organizations, or those of the publisher, the editors and the reviewers. Any product that may be evaluated in this article, or claim that may be made by its manufacturer, is not guaranteed or endorsed by the publisher.

## Supplementary material

Supplementary Figure 1 IGF2BP3 overexpression enhanced GBC proliferation and migration **(A, B)** OCUG-1 cells were transfected with PLVX-IGF2BP3 and control plasmids, and effect of the transfection was verified by qPCR **(A)** and western blot **(B, C–E)** Cell growth ability was detected by performing CCK8 assay **(C)**, colony information assay **(D)** and EdU assay (**E**, 100X, Hoechst (blue), Edu (red), scale bar: 100μm) **(F)** Transwell assays were performed to measure the migration ability in treated OUCG-1 cells (40X, scale bar: 50μm).

Supplementary Figure 2 Statistical analysis of the results from transwell assays.

Supplementary Figure 3 Gel electrophoresis showing the result of RIP-qPCR.

Supplementary Figure 4 Western blotting of downstream pathway targets in GBC cells with IGF2BP3 depletion.
