# Supplementary figures and images for: IGF2BP3 promotes progression of gallbladder carcinoma by stabilizing KLK5 mRNA in N6-methyladenosine-dependent binding
Source: Front Oncol. 2022 Oct 13;12:1035871. doi: 10.3389/fonc.2022.1035871 (PMC9606626; doi:10.3389/fonc.2022.1035871)

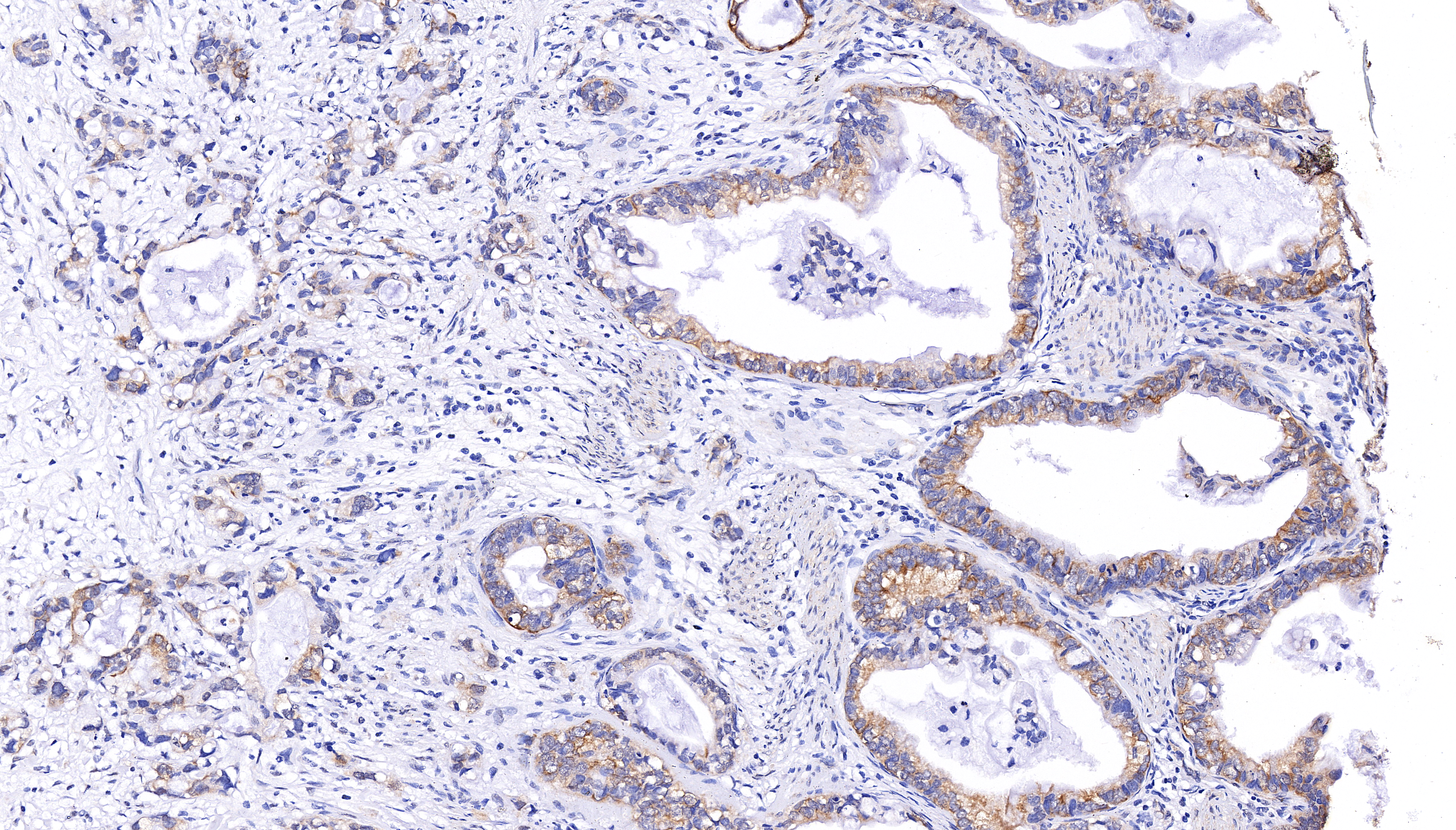

Supplement: Supplementary Figure 1 — IGF2BP3 overexpression enhanced GBC proliferation and migration (A, B) OCUG-1 cells were transfected with PLVX-IGF2BP3 and control plasmids, and effect of the transfection was verified by qPCR (A) and western blot (B, C–E) Cell growth ability was detected by performing CCK8 assay (C), colony information assay (D) and EdU assay (E, 100X, Hoechst (blue), Edu (red), scale bar: 100μm) (F) Transwell assays were performed to measure the migration ability in treated OUCG-1 cells (40X, scale bar: 50μm). [file DataSheet_1.zip › Additional Images/Image 10.TIF]

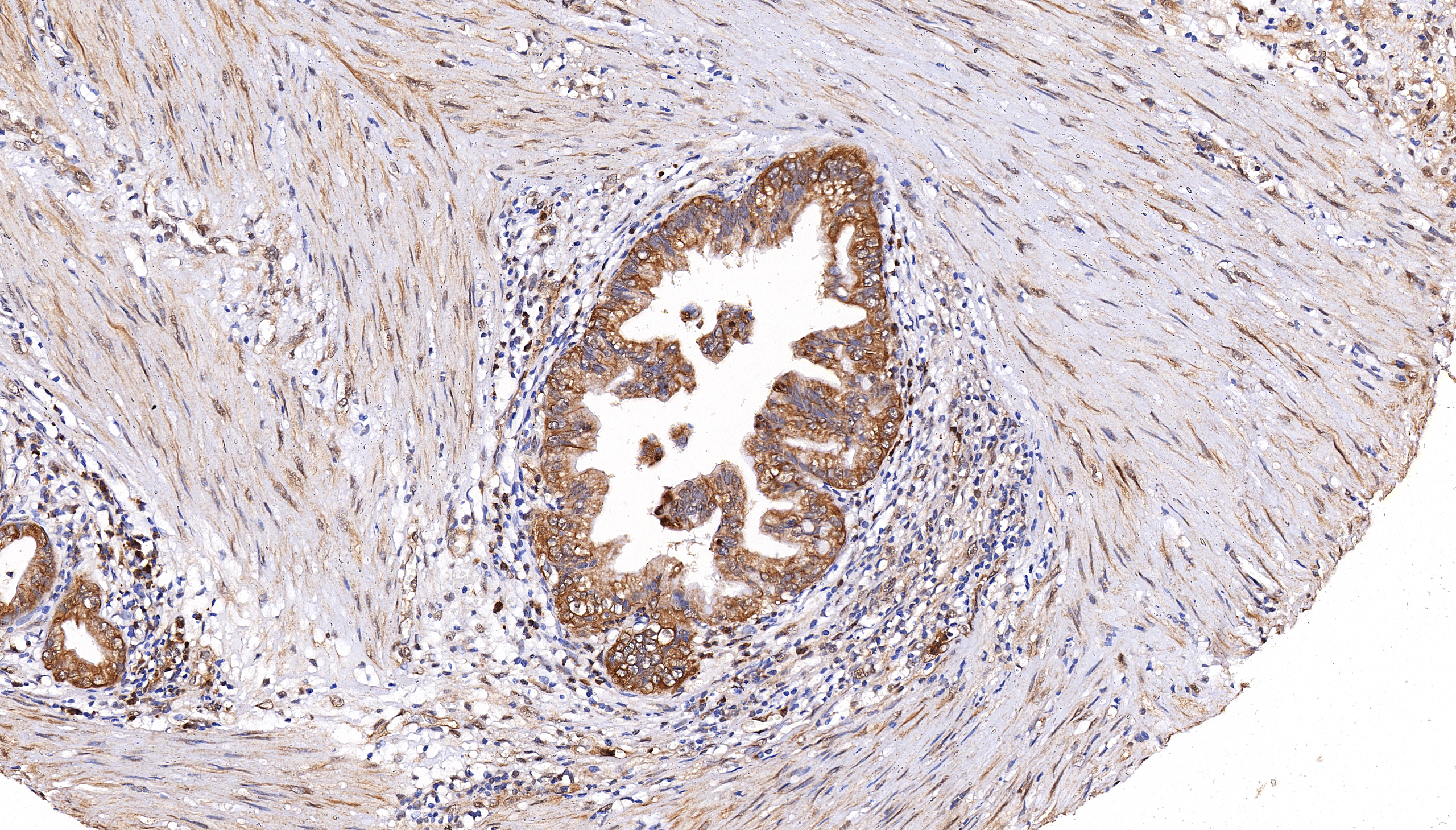

Supplement: Supplementary Figure 1 — IGF2BP3 overexpression enhanced GBC proliferation and migration (A, B) OCUG-1 cells were transfected with PLVX-IGF2BP3 and control plasmids, and effect of the transfection was verified by qPCR (A) and western blot (B, C–E) Cell growth ability was detected by performing CCK8 assay (C), colony information assay (D) and EdU assay (E, 100X, Hoechst (blue), Edu (red), scale bar: 100μm) (F) Transwell assays were performed to measure the migration ability in treated OUCG-1 cells (40X, scale bar: 50μm). [file DataSheet_1.zip › Additional Images/Image 11.TIF]

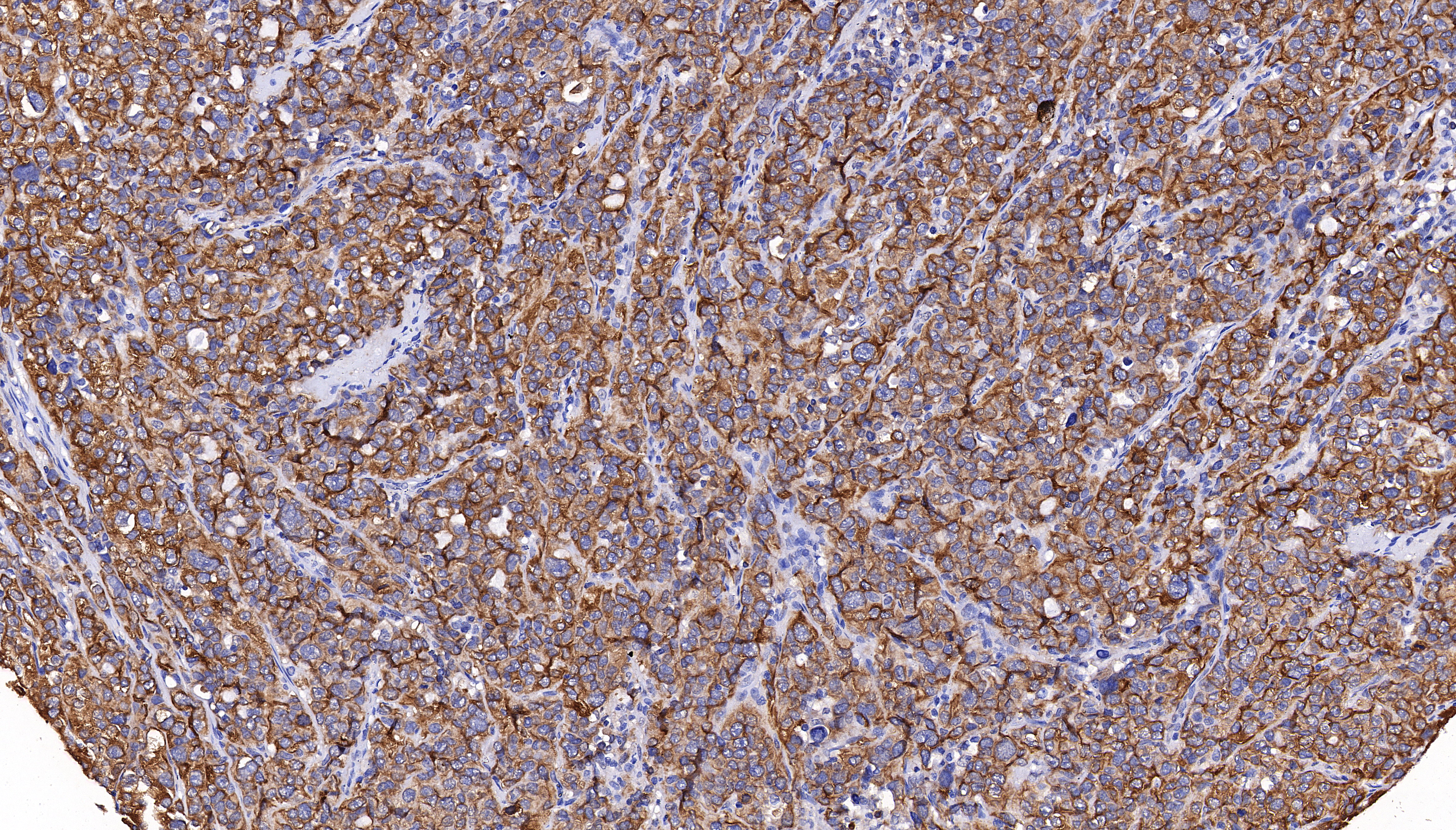

Supplement: Supplementary Figure 1 — IGF2BP3 overexpression enhanced GBC proliferation and migration (A, B) OCUG-1 cells were transfected with PLVX-IGF2BP3 and control plasmids, and effect of the transfection was verified by qPCR (A) and western blot (B, C–E) Cell growth ability was detected by performing CCK8 assay (C), colony information assay (D) and EdU assay (E, 100X, Hoechst (blue), Edu (red), scale bar: 100μm) (F) Transwell assays were performed to measure the migration ability in treated OUCG-1 cells (40X, scale bar: 50μm). [file DataSheet_1.zip › Additional Images/Image 12.TIF]

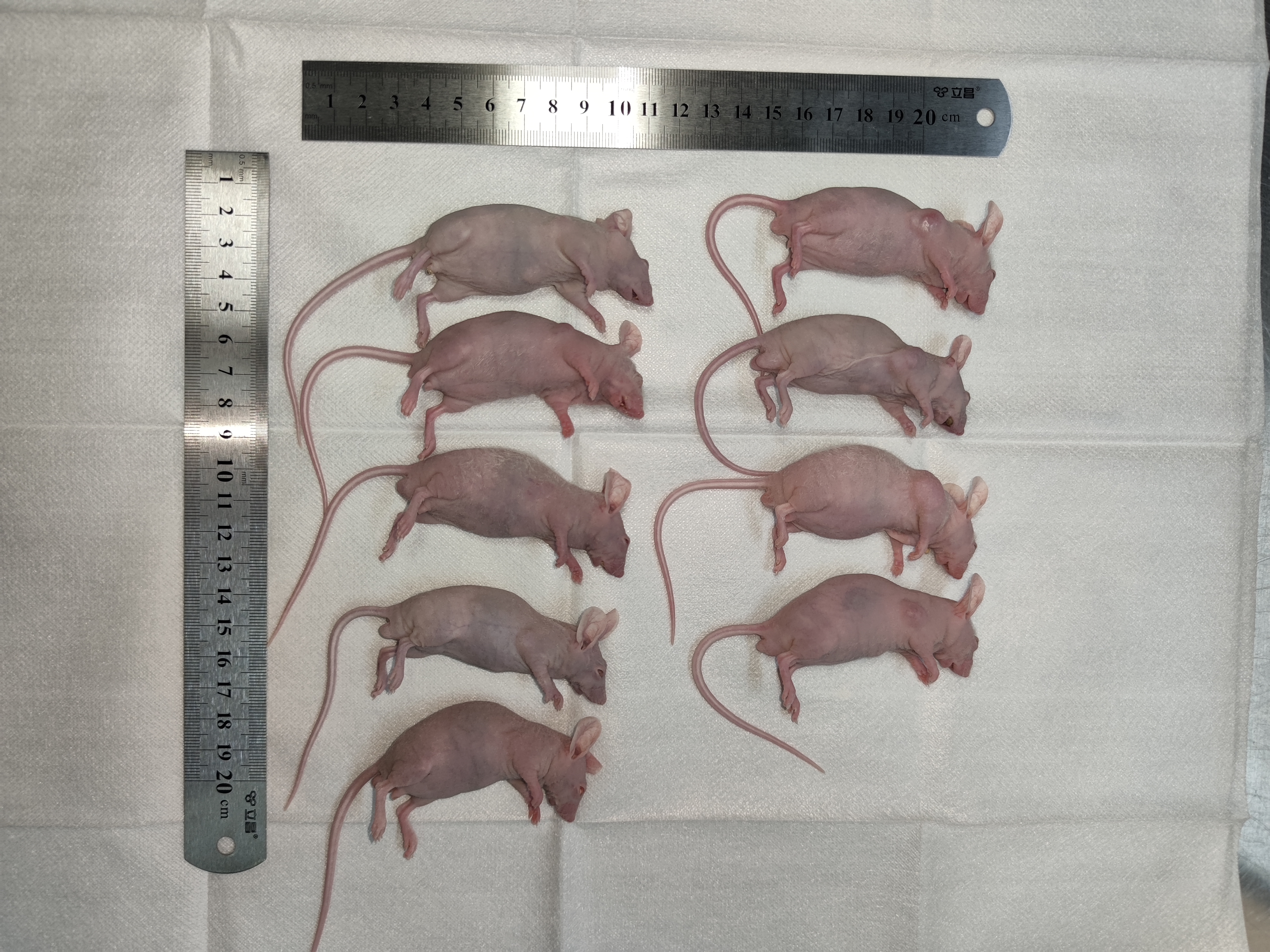

Supplement: Supplementary Figure 1 — IGF2BP3 overexpression enhanced GBC proliferation and migration (A, B) OCUG-1 cells were transfected with PLVX-IGF2BP3 and control plasmids, and effect of the transfection was verified by qPCR (A) and western blot (B, C–E) Cell growth ability was detected by performing CCK8 assay (C), colony information assay (D) and EdU assay (E, 100X, Hoechst (blue), Edu (red), scale bar: 100μm) (F) Transwell assays were performed to measure the migration ability in treated OUCG-1 cells (40X, scale bar: 50μm). [file DataSheet_1.zip › Additional Images/Image 13.TIF]

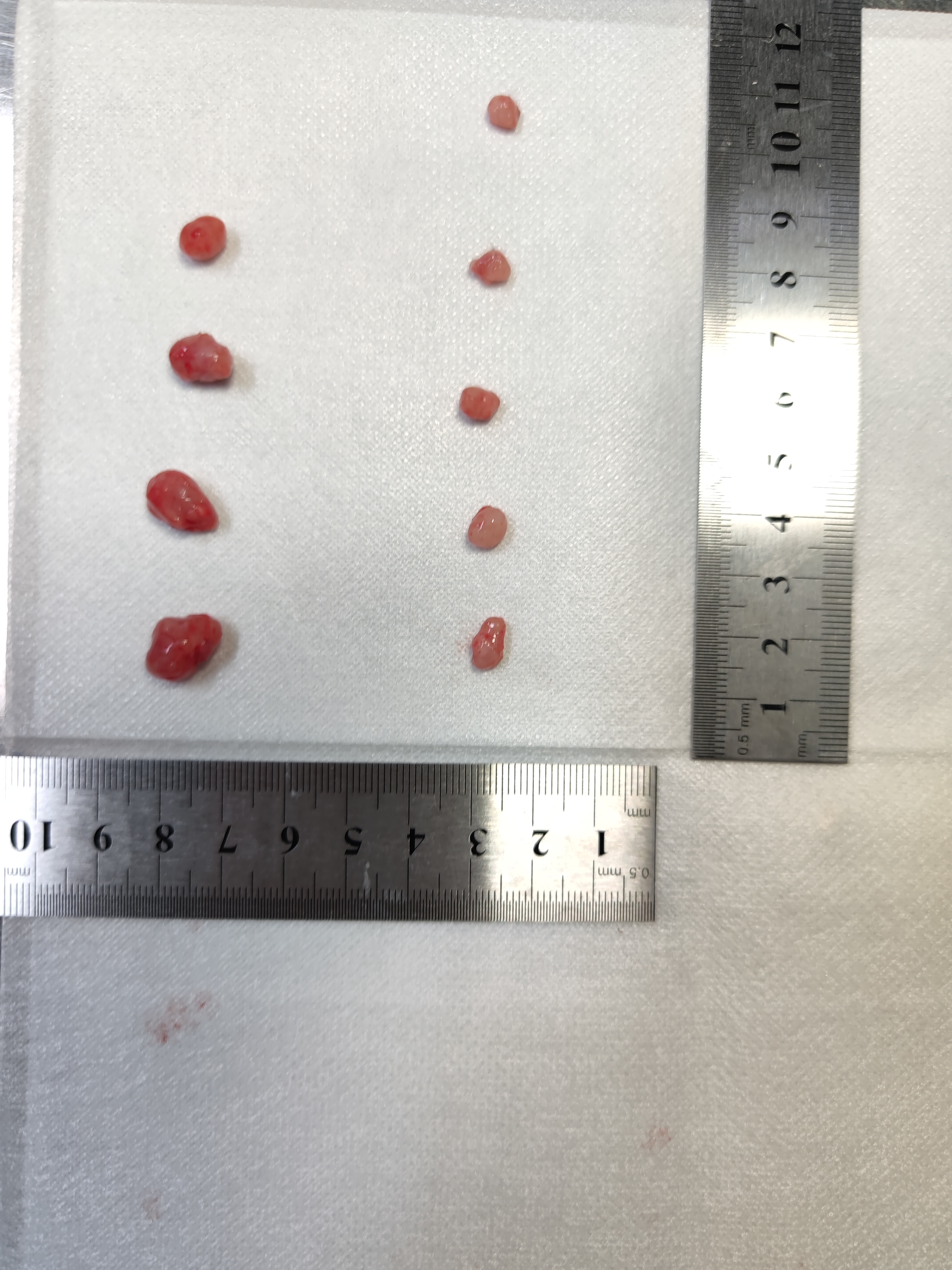

Supplement: Supplementary Figure 1 — IGF2BP3 overexpression enhanced GBC proliferation and migration (A, B) OCUG-1 cells were transfected with PLVX-IGF2BP3 and control plasmids, and effect of the transfection was verified by qPCR (A) and western blot (B, C–E) Cell growth ability was detected by performing CCK8 assay (C), colony information assay (D) and EdU assay (E, 100X, Hoechst (blue), Edu (red), scale bar: 100μm) (F) Transwell assays were performed to measure the migration ability in treated OUCG-1 cells (40X, scale bar: 50μm). [file DataSheet_1.zip › Additional Images/Image 14.TIF]

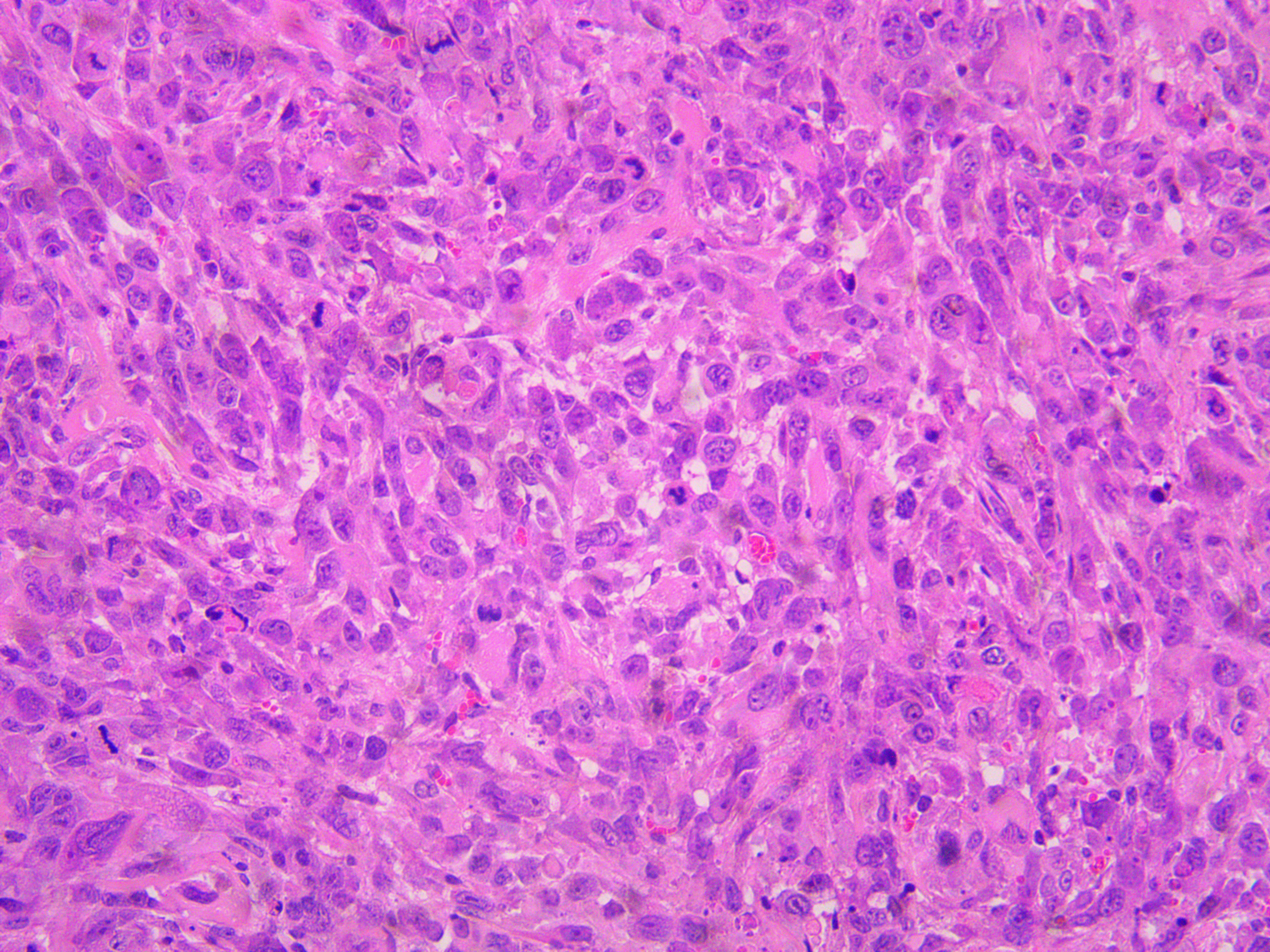

Supplement: Supplementary Figure 1 — IGF2BP3 overexpression enhanced GBC proliferation and migration (A, B) OCUG-1 cells were transfected with PLVX-IGF2BP3 and control plasmids, and effect of the transfection was verified by qPCR (A) and western blot (B, C–E) Cell growth ability was detected by performing CCK8 assay (C), colony information assay (D) and EdU assay (E, 100X, Hoechst (blue), Edu (red), scale bar: 100μm) (F) Transwell assays were performed to measure the migration ability in treated OUCG-1 cells (40X, scale bar: 50μm). [file DataSheet_1.zip › Additional Images/Image 15.TIF]

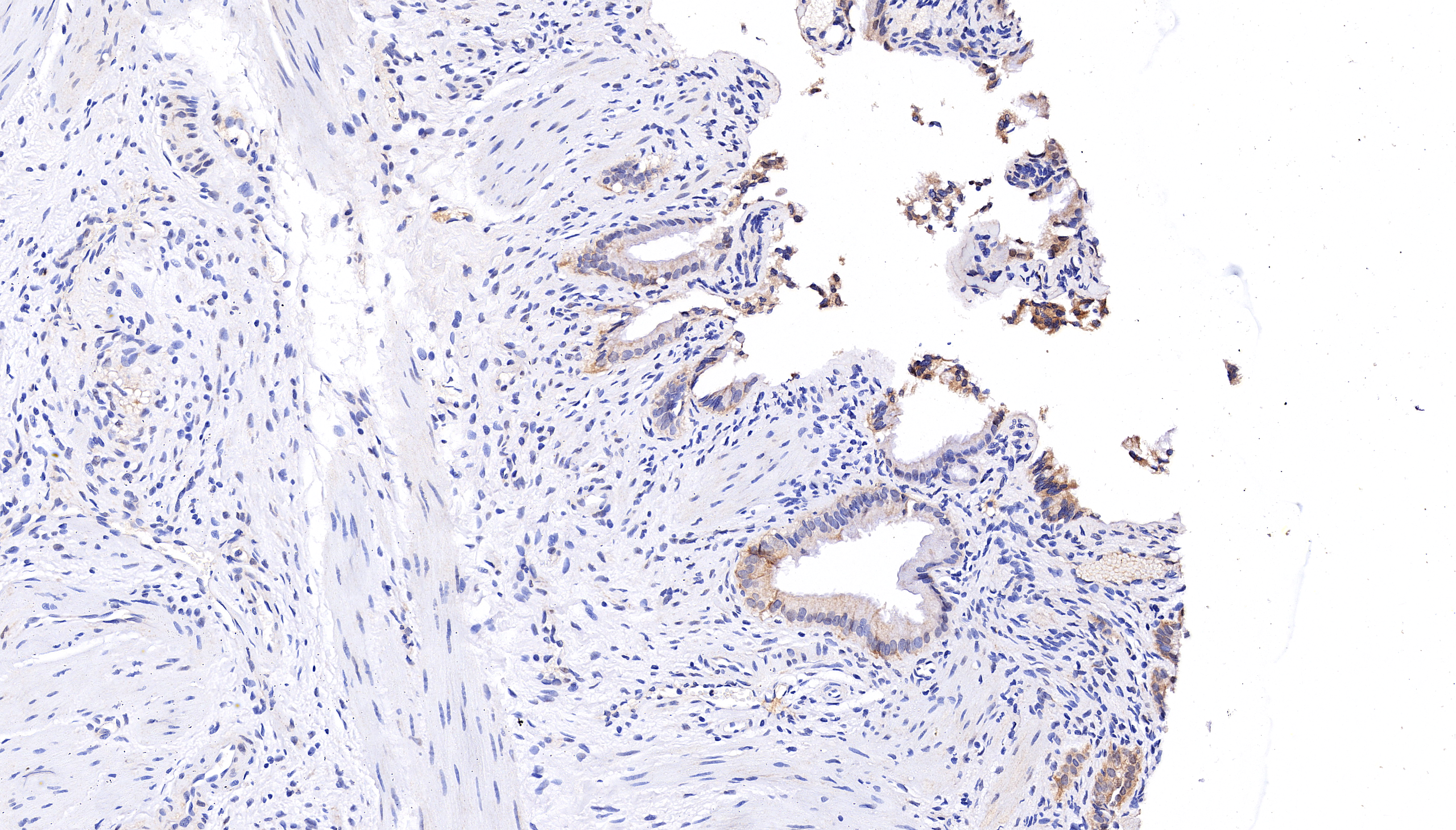

Supplement: Supplementary Figure 1 — IGF2BP3 overexpression enhanced GBC proliferation and migration (A, B) OCUG-1 cells were transfected with PLVX-IGF2BP3 and control plasmids, and effect of the transfection was verified by qPCR (A) and western blot (B, C–E) Cell growth ability was detected by performing CCK8 assay (C), colony information assay (D) and EdU assay (E, 100X, Hoechst (blue), Edu (red), scale bar: 100μm) (F) Transwell assays were performed to measure the migration ability in treated OUCG-1 cells (40X, scale bar: 50μm). [file DataSheet_1.zip › Additional Images/Image 5.TIF]

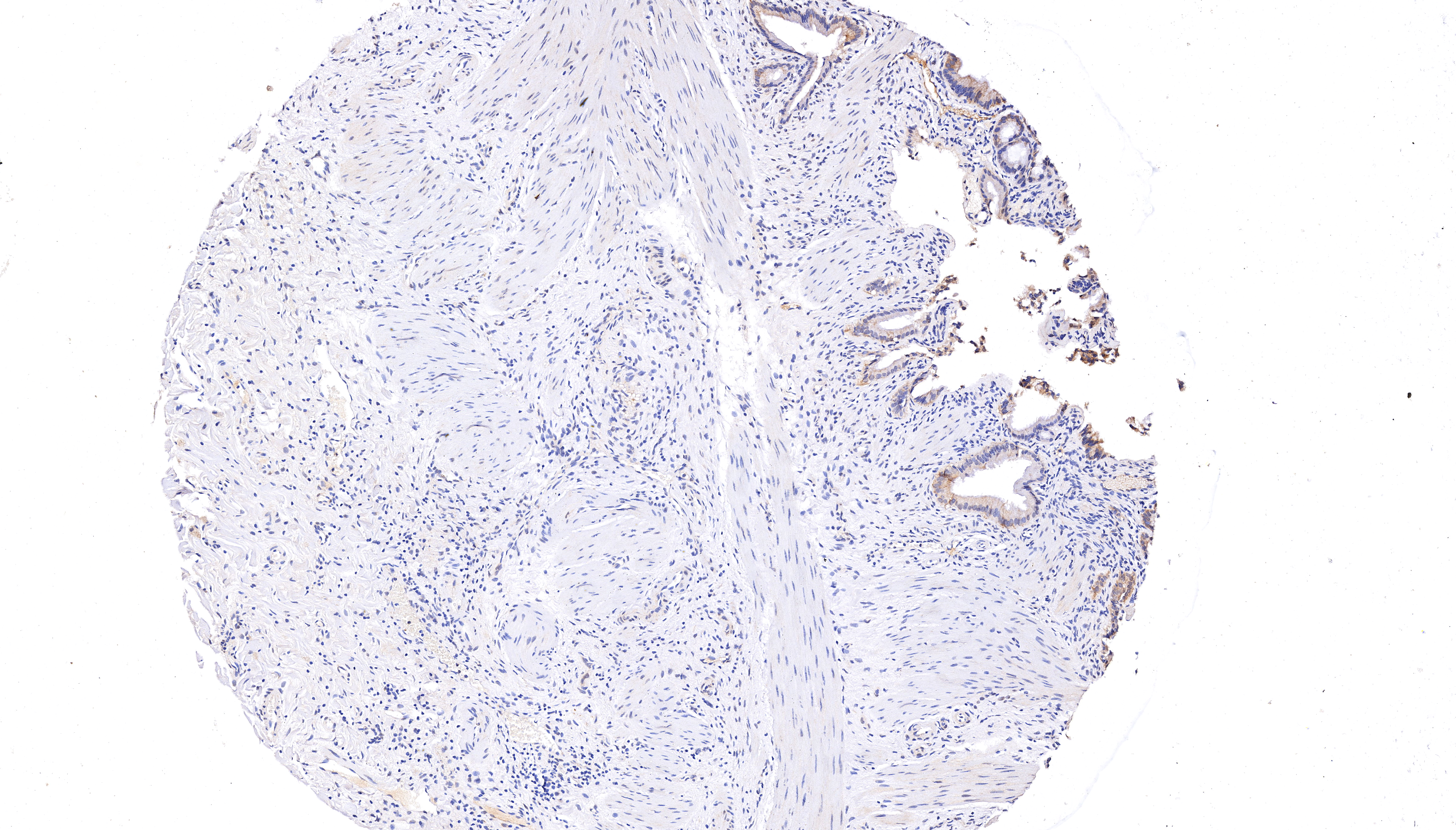

Supplement: Supplementary Figure 1 — IGF2BP3 overexpression enhanced GBC proliferation and migration (A, B) OCUG-1 cells were transfected with PLVX-IGF2BP3 and control plasmids, and effect of the transfection was verified by qPCR (A) and western blot (B, C–E) Cell growth ability was detected by performing CCK8 assay (C), colony information assay (D) and EdU assay (E, 100X, Hoechst (blue), Edu (red), scale bar: 100μm) (F) Transwell assays were performed to measure the migration ability in treated OUCG-1 cells (40X, scale bar: 50μm). [file DataSheet_1.zip › Additional Images/Image 6.TIF]

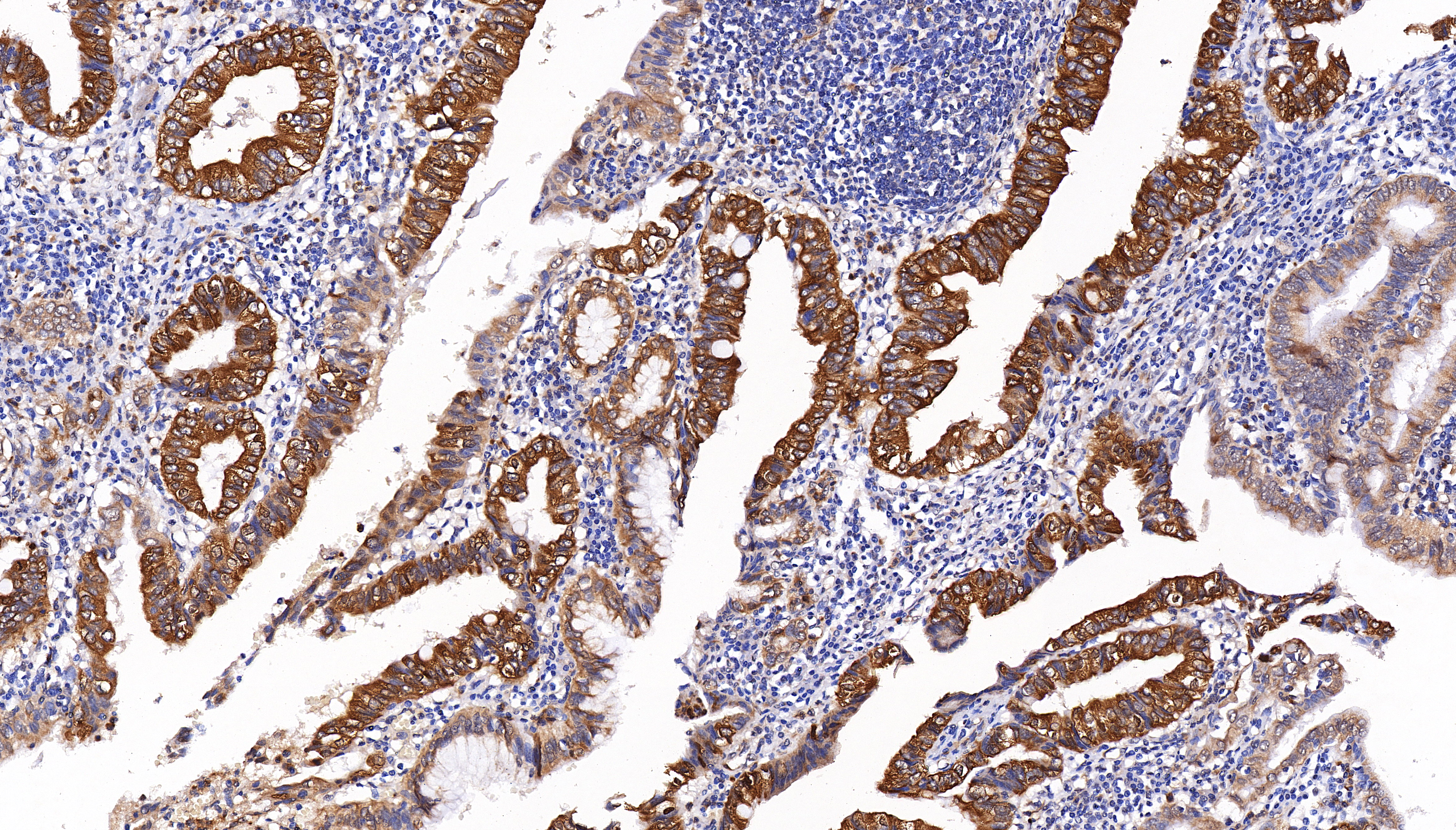

Supplement: Supplementary Figure 1 — IGF2BP3 overexpression enhanced GBC proliferation and migration (A, B) OCUG-1 cells were transfected with PLVX-IGF2BP3 and control plasmids, and effect of the transfection was verified by qPCR (A) and western blot (B, C–E) Cell growth ability was detected by performing CCK8 assay (C), colony information assay (D) and EdU assay (E, 100X, Hoechst (blue), Edu (red), scale bar: 100μm) (F) Transwell assays were performed to measure the migration ability in treated OUCG-1 cells (40X, scale bar: 50μm). [file DataSheet_1.zip › Additional Images/Image 7.TIF]

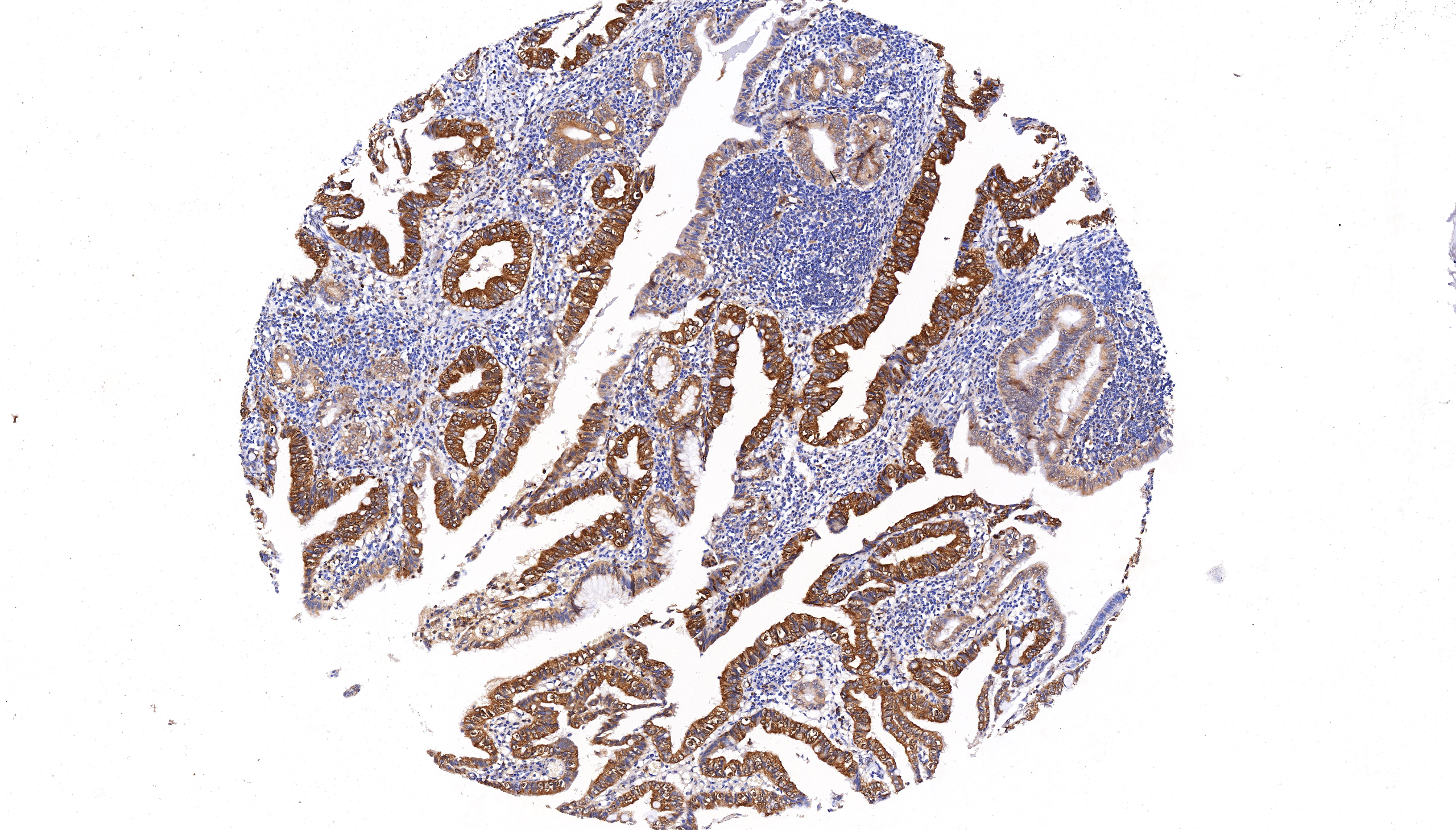

Supplement: Supplementary Figure 1 — IGF2BP3 overexpression enhanced GBC proliferation and migration (A, B) OCUG-1 cells were transfected with PLVX-IGF2BP3 and control plasmids, and effect of the transfection was verified by qPCR (A) and western blot (B, C–E) Cell growth ability was detected by performing CCK8 assay (C), colony information assay (D) and EdU assay (E, 100X, Hoechst (blue), Edu (red), scale bar: 100μm) (F) Transwell assays were performed to measure the migration ability in treated OUCG-1 cells (40X, scale bar: 50μm). [file DataSheet_1.zip › Additional Images/Image 8.TIF]

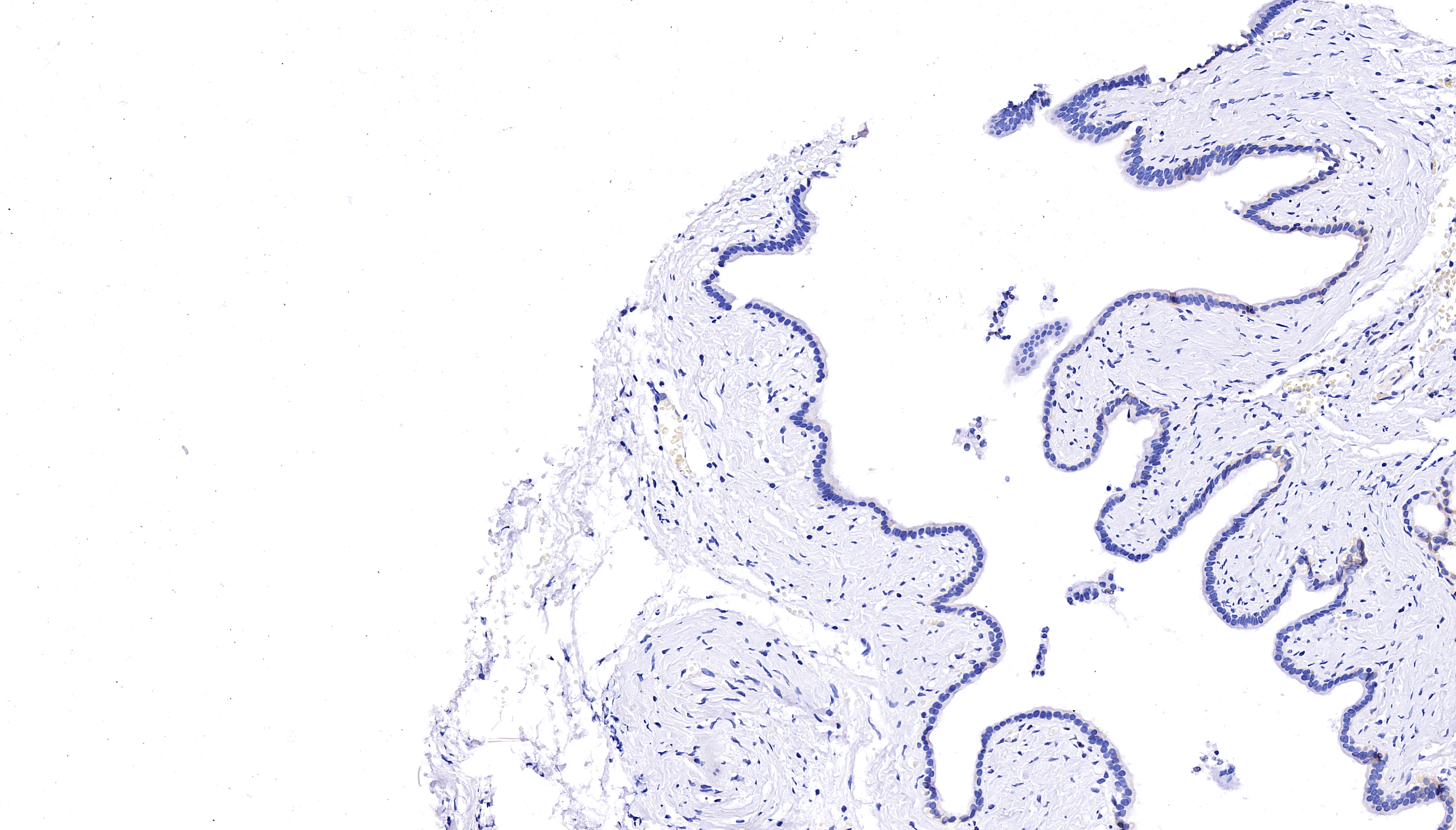

Supplement: Supplementary Figure 1 — IGF2BP3 overexpression enhanced GBC proliferation and migration (A, B) OCUG-1 cells were transfected with PLVX-IGF2BP3 and control plasmids, and effect of the transfection was verified by qPCR (A) and western blot (B, C–E) Cell growth ability was detected by performing CCK8 assay (C), colony information assay (D) and EdU assay (E, 100X, Hoechst (blue), Edu (red), scale bar: 100μm) (F) Transwell assays were performed to measure the migration ability in treated OUCG-1 cells (40X, scale bar: 50μm). [file DataSheet_1.zip › Additional Images/Image 9.TIF]

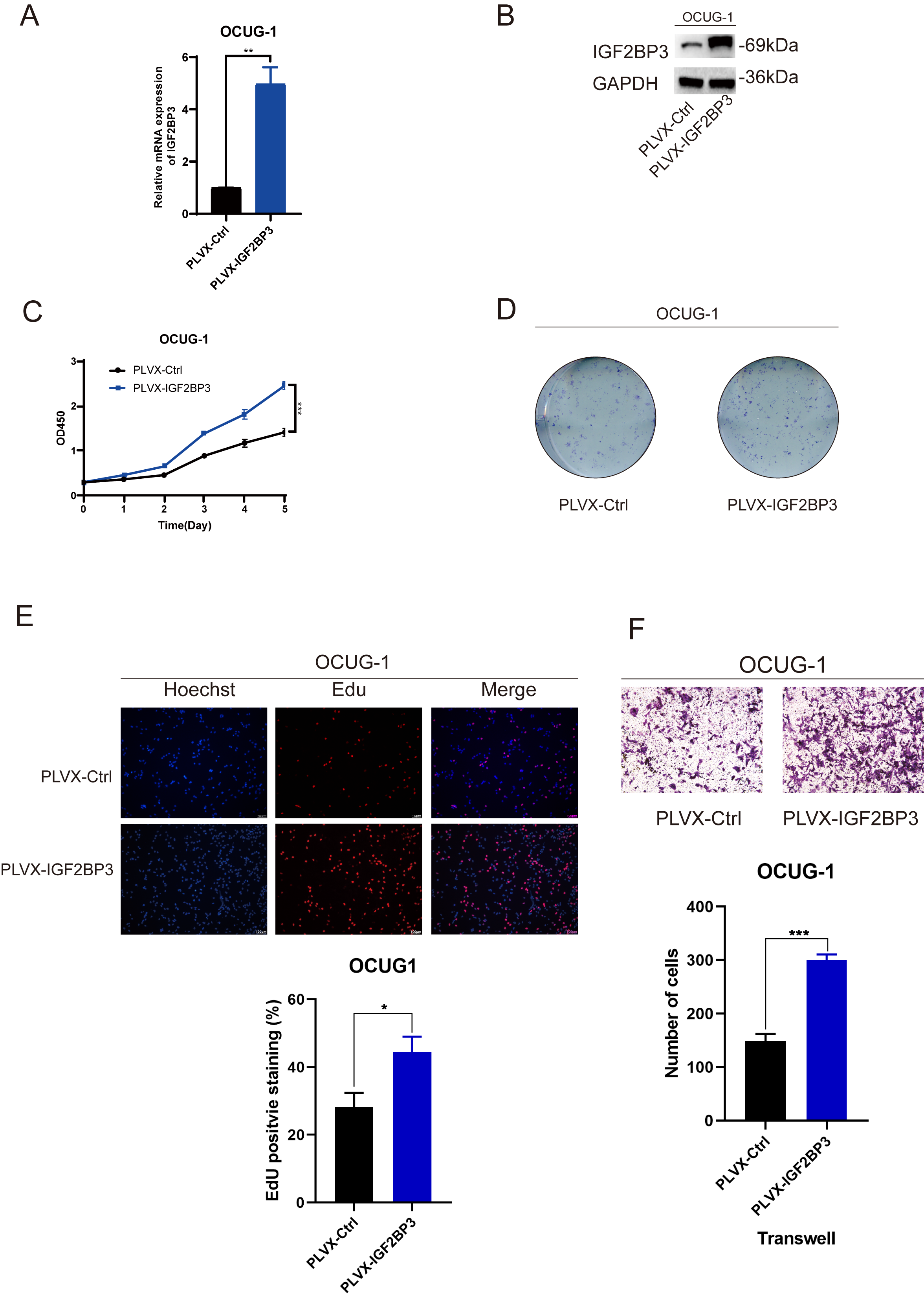

Supplement: Supplementary Figure 1 — IGF2BP3 overexpression enhanced GBC proliferation and migration (A, B) OCUG-1 cells were transfected with PLVX-IGF2BP3 and control plasmids, and effect of the transfection was verified by qPCR (A) and western blot (B, C–E) Cell growth ability was detected by performing CCK8 assay (C), colony information assay (D) and EdU assay (E, 100X, Hoechst (blue), Edu (red), scale bar: 100μm) (F) Transwell assays were performed to measure the migration ability in treated OUCG-1 cells (40X, scale bar: 50μm). [file DataSheet_1.zip › Supplementary Figure S1.TIF]

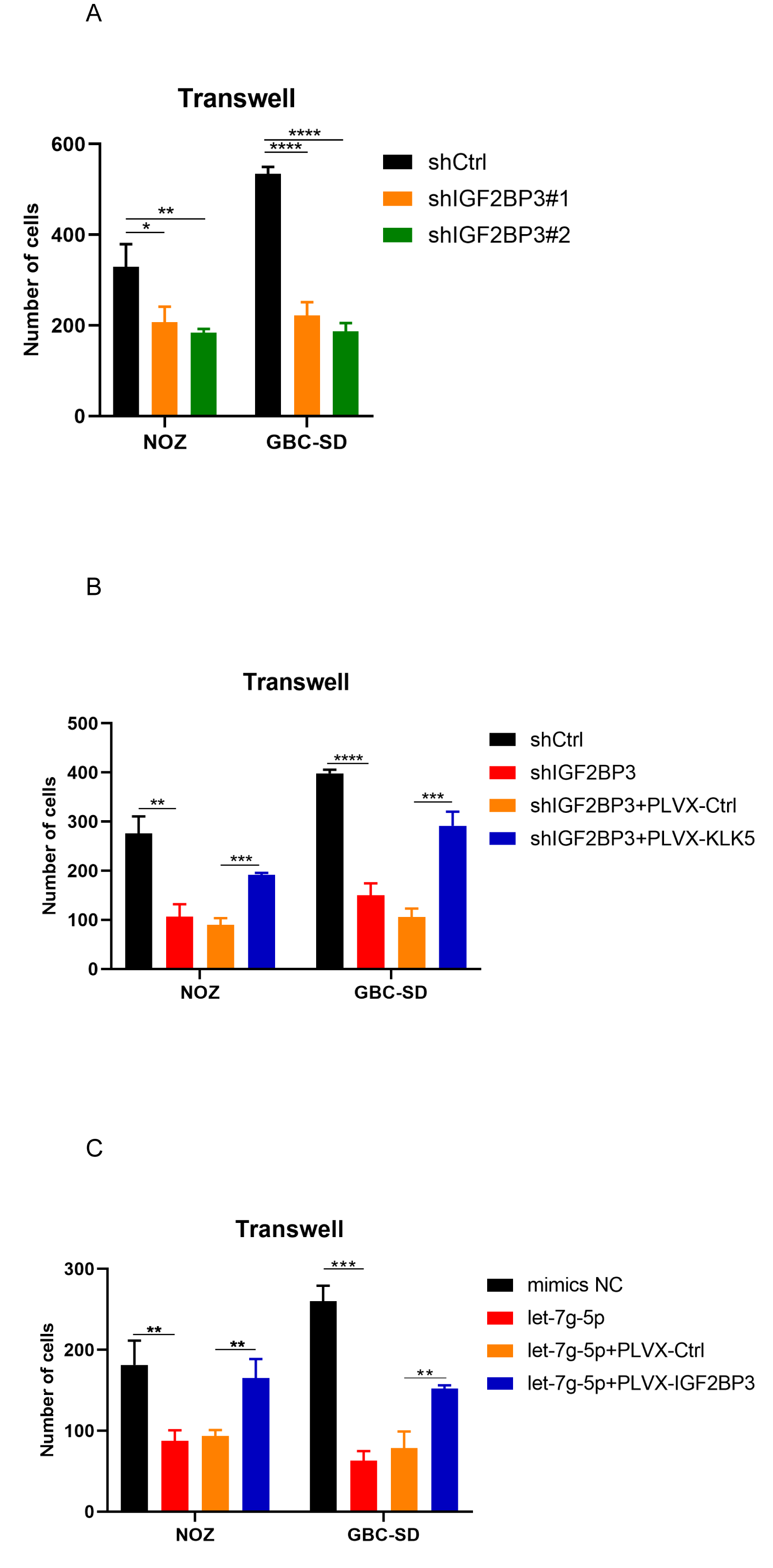

Supplement: Supplementary Figure 1 — IGF2BP3 overexpression enhanced GBC proliferation and migration (A, B) OCUG-1 cells were transfected with PLVX-IGF2BP3 and control plasmids, and effect of the transfection was verified by qPCR (A) and western blot (B, C–E) Cell growth ability was detected by performing CCK8 assay (C), colony information assay (D) and EdU assay (E, 100X, Hoechst (blue), Edu (red), scale bar: 100μm) (F) Transwell assays were performed to measure the migration ability in treated OUCG-1 cells (40X, scale bar: 50μm). [file DataSheet_1.zip › Supplementary Figure S2.TIF]

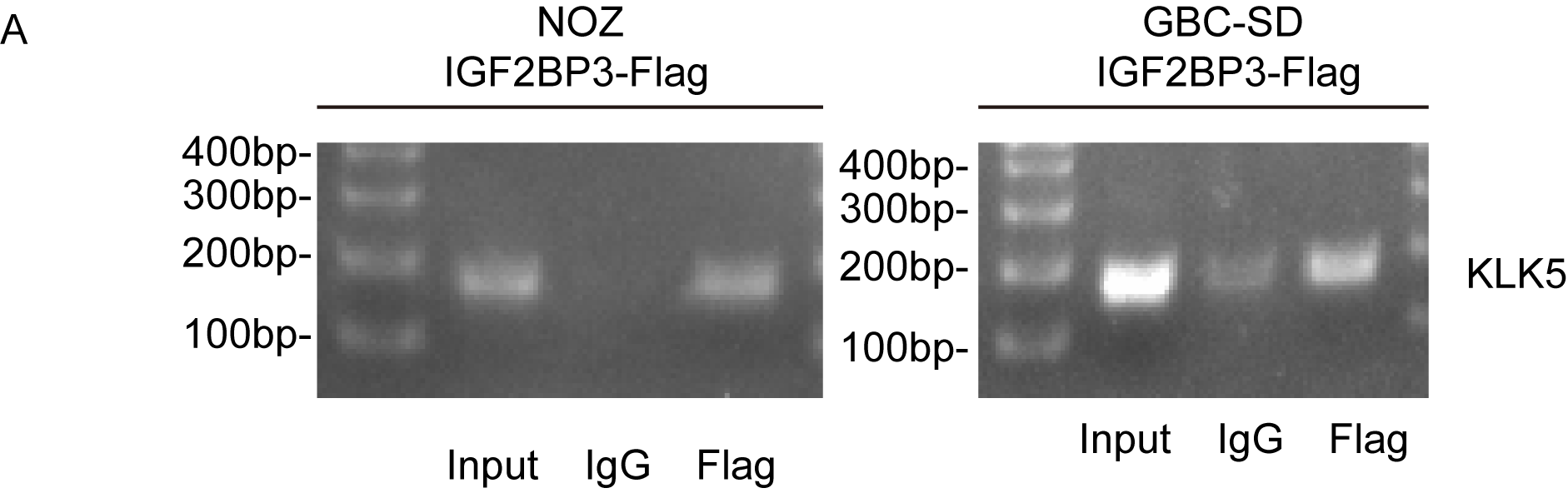

Supplement: Supplementary Figure 1 — IGF2BP3 overexpression enhanced GBC proliferation and migration (A, B) OCUG-1 cells were transfected with PLVX-IGF2BP3 and control plasmids, and effect of the transfection was verified by qPCR (A) and western blot (B, C–E) Cell growth ability was detected by performing CCK8 assay (C), colony information assay (D) and EdU assay (E, 100X, Hoechst (blue), Edu (red), scale bar: 100μm) (F) Transwell assays were performed to measure the migration ability in treated OUCG-1 cells (40X, scale bar: 50μm). [file DataSheet_1.zip › Supplementary Figure S3.TIF]

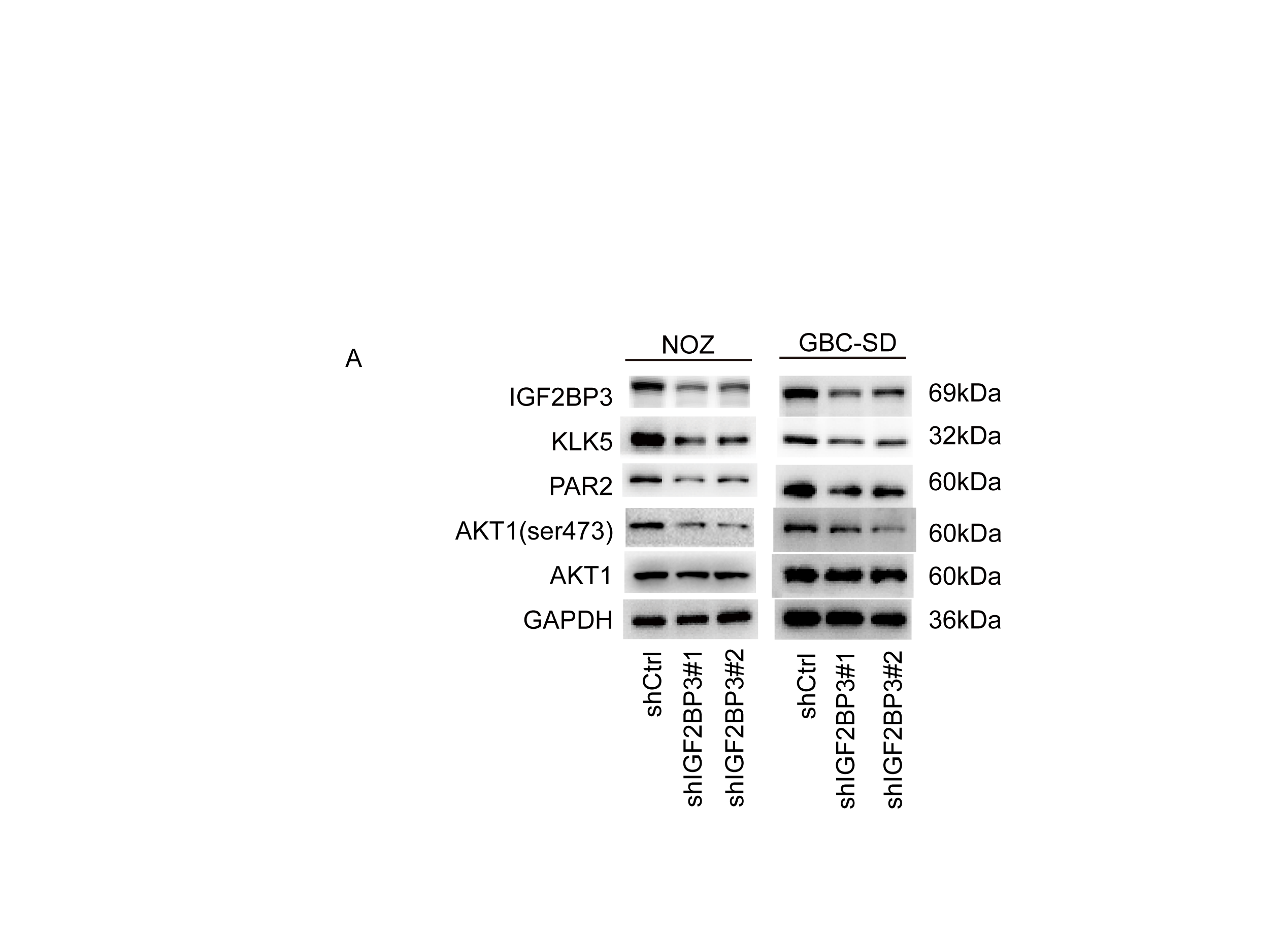

Supplement: Supplementary Figure 1 — IGF2BP3 overexpression enhanced GBC proliferation and migration (A, B) OCUG-1 cells were transfected with PLVX-IGF2BP3 and control plasmids, and effect of the transfection was verified by qPCR (A) and western blot (B, C–E) Cell growth ability was detected by performing CCK8 assay (C), colony information assay (D) and EdU assay (E, 100X, Hoechst (blue), Edu (red), scale bar: 100μm) (F) Transwell assays were performed to measure the migration ability in treated OUCG-1 cells (40X, scale bar: 50μm). [file DataSheet_1.zip › Supplementary Figure S4.TIF]
